# Supplementary material for: DPP-4 inhibitors sitagliptin and PF-00734,200 mitigate dopaminergic neurodegeneration, neuroinflammation and behavioral impairment in the rat 6-OHDA model of Parkinson’s disease
Source: GeroScience. 2024 Apr 2;46(5):4349–71. doi: 10.1007/s11357-024-01116-0 (PMC11336009; doi:10.1007/s11357-024-01116-0)
Supplement: Supplementary file 1 — Supplementary file1 (DOCX 14214 KB) [file 11357_2024_1116_MOESM1_ESM.docx]

**DPP-4 inhibitors sitagliptin and PF-00734,200 mitigate dopaminergic neurodegeneration, neuroinflammation and behavioral impairment in the rat 6-OHDA model of Parkinson’s disease**

^1^Seong-Jin Yu, ^1,2^Yun Wang*, ^2^Hui Shen, ^1^Eun-Kyung Bae, ^3^Yazhou Li, ^4^Kumar Sambamurti, ^5^Michael A Tones^♦^, ^6^Margaret M Zaleska^♦^, ^7^Barry J Hoffer, ^3^Nigel H Greig*

1. Center for Neuropsychiatric Research, National Health Research Institutes, Zhunan, Taiwan 35053
2. National Institute on Drug Abuse, Intramural Research Program, National Institutes of Health, Baltimore, MD 21224, USA
3. National Institute on Aging, Intramural Research Program, National Institutes of Health, Baltimore, MD 21224, USA
4. Department of Neurosciences, the Medical University of South Carolina, Charleston, SC 29425, USA.
5. Cadre Bioscience, Saint Louis, MO 63110, USA.
6. Neuro-D Consulting LLC, Penn Valley, PA 19072, USA.
7. Department of Neurosurgery, University Hospitals of Cleveland, Case Western Reserve University School of Medicine, Cleveland, OH 44106, USA.

*Co-corresponding authors: Nigel H. Greig: [Greign@grc.nia.nih.gov](mailto:Greign@grc.nia.nih.gov) &

Yun Wang: [ywang@nhri.edu.tw](mailto:ywang@nhri.edu.tw)

^♦^At the time of initiation of this project, MAT and MMZ were employees of Pfizer Inc.

Seong-Jin Yu: [b7508@nhri.edu.tw](mailto:b7508@nhri.edu.tw)

Yun Wang: [ywang@nhri.edu.tw](mailto:ywang@nhri.edu.tw)

Hui Shen: [HShen@intra.nida.nih.gov](mailto:HShen@intra.nida.nih.gov)

Eun-Kyung Bae: [baee@nhri.edu.tw](mailto:baee@nhri.edu.tw)

Yazhou Li: [liyaz@mail.nih.gov](mailto:liyaz@mail.nih.gov)

Kumar Sambamurti: [sambak@musc.edu](mailto:sambak@musc.edu)

Mike A. Tones: [mike.tones@cadrebioscience.com](mailto:mike.tones@cadrebioscience.com)

Margaret M. Zaleska: [margaret.m.zaleska@gmail.com](mailto:margaret.m.zaleska@gmail.com)

Barry J. Hoffer: [bjh82@case.edu](mailto:bjh82@case.edu)

Nigel H. Greig: [Greign@grc.nia.nih.gov](mailto:Greign@grc.nia.nih.gov)

Grants:

1) Michael J Fox Foundation

2) Intramural Research Program, NIA, NIH (AG000333)

3) National Health Research Institutes, Taiwan (Intramural grant)

4) MOST-110-2320-B-400-007 (Ministry of Science and Technology, Taiwan)

5) MOST-111-2320-B-400-010 (Ministry of Science and Technology, Taiwan)

**Abstract**

Epidemiological studies report an elevated risk of Parkinson's disease (PD) in patients with type 2 diabetes mellitus (T2DM) that is mitigated in those prescribed dipeptidyl peptidase 4 (DPP-4) inhibitors. With an objective to characterize clinically translatable doses of DPP-4 inhibitors (gliptins) in a well-characterized PD rodent model, sitagliptin, PF-00734,200 or vehicle were orally administered to rats initiated either 7-days before or 7-days after unilateral medial forebrain bundle 6-hydroxydopamine (6-OHDA) lesioning. Measures of dopaminergic cell viability, dopamine content, neuroinflammation and neurogenesis were evaluated thereafter in ipsi- and contralateral brain. Plasma and brain incretin and DPP-4 activity levels were quantified. Furthermore, brain incretin receptor levels were age-dependently evaluated in rodents, in 6-OHDA challenged animals and human subjects with/without PD. Cellular studies evaluated neurotrophic/neuroprotective actions of combined incretin administration. Pre-treatment with oral sitagliptin or PF-00734,200 reduced methamphetamine (meth)-induced rotation post-lesioning and dopaminergic degeneration in lesioned substantia nigra pars compacta (SNc) and striatum. Direct intracerebroventricular gliptin administration lacked neuroprotective actions, indicating that systemic incretin-mediated mechanisms underpin gliptin-induced favorable brain effects. Post-treatment with a 3-fold higher oral gliptin dose, likewise, mitigated meth-induced rotation, dopaminergic neurodegeneration and neuroinflammation, and augmented neurogenesis. These gliptin-induced actions associated with 70-80% plasma and 20-30% brain DPP-4 inhibition, and elevated plasma and brain incretin levels. Brain incretin receptor protein levels were age-dependently maintained in rodents, preserved in rats challenged with 6-OHDA, and in humans with PD. Combined GLP-1 and GIP receptor activation in neuronal cultures resulted in neurotrophic/neuroprotective actions superior to single agonists alone. In conclusion, these studies support further evaluation of the repurposing of clinically approved gliptins as a treatment strategy for PD.

**Keywords:** Gliptins; sitagliptin; PF-00734,200; Parkinson’s disease; 6-hydroxydopamine; dopamine; neuroinflammation; neurodegeneration; neurogenesis; incretins, GLP-1; GIP.

**Introduction**

Parkinson's Disease (PD) is the second most prevalent progressive neurodegenerative disorder worldwide, and advancing age is its greatest risk factor [1]. PD impacts in excess of 8.5 million people [1], and is characterized by a loss of dopamine (DA)-generating neurons within the substantia nigra pars compacta (SNc) and resulting deficits in the nigrostriatal dopaminergic pathway [2]. This leads to DA deficiency in the caudate nucleus and putamen, subsequent loss of motor coordination, neuroinflammation and various non-motor symptoms. Existing PD treatments are chiefly focused to pharmacologically restore DA to mitigate motor symptoms, and provide symptomatic relief during the early disease course [3]. Such treatments do not, however, halt or reverse neuronal loss, nor do they modify disease progression. Hence, new strategies to mitigate PD development are eagerly required and, in this regard, the repositioning of a well-tolerated and efficacious clinically approved drug from one disease to another whose underlying mechanisms have commonality represents a potentially rapid and efficient means of drug development.

The focus of the current study is to evaluate the potential of dipeptidyl peptidase-4 (DPP-4) inhibitors, also known as the gliptin drug class, as a new treatment strategy for PD, since standard PD pharmacotherapy is limited by a progressive loss of efficacy and development of adverse effects with time. Gliptins are approved and widely used in the effective treatment of type 2 diabetes mellitus (T2DM) [4, 5]. DPP-4 is the key enzyme responsible for the metabolism of the endogenous incretins, glucagon-like peptide-1 (GLP-1) and glucose-dependent insulinotropic polypeptide (GIP) [4, 5]. These peptides are generated and released from the L and K cells of the small intestine, respectively, following food ingestion [6-8], and bind to their cognate receptors (GLP-1R and GIP-R) on pancreatic β-cells. Receptor stimulation induces activation of adenylyl cyclase, cyclic adenosine monophosphate (cAMP) accumulation, protein kinase A (PKA) activation and release of insulin to regulate blood glucose levels [6-8]. Incretin-mediated insulin release is “glucose dependent” and occurs only when blood glucose levels are elevated [9, 10]. Importantly, these incretins additionally provide trophic and protective actions on pancreatic β-cells [4-9].

The GLP-1R and GIP-R are also present in a number of other organs, including the brain - where they are highly expressed across a number of neurons [11-13], and can respond to both GLP-1 and GIP, which readily enter the brain [11, 14-16]. The activation of the GLP-1R on neurons induces potent neurotrophic and neuroprotective actions in cellular and animal models of neural injury and neurodegeneration [13, 17-19], including PD [18, 20-25]. Furthermore, incretin receptors are reported to be present on activated microglia and astrocytes, and their activation quells neuroinflammation [17-19, 22, 25]. The DPP-4 resistant long-acting GLP-1 mimetic Exendin-4, approved for the treatment of T2DM, is hence being clinically assessed in neurological disorders [20-25] and has demonstrated promising efficacy in clinical studies when administered to PD patients by twice daily [26, 27] or once weekly injection [28-30].

An alternative strategy to activate GLP-1Rs in brain to achieve therapeutic benefit is to elevate physiological incretin levels by inhibiting DPP-4, the enzyme responsible for incretin inactivation. Gliptins are well tolerated, US FDA approved, effectively used in the treatment of T2DM and, importantly, likewise have glucose-dependent actions. Hence, they are not associated with the induction of hypoglycemia when administered to euglycemic subjects [4, 5]. Notably, DPP-4 inhibitors are oral drugs and offer the additional potential advantage of augmenting both GLP-1 and GIP actions to provide synergistic effects [31, 32].

As presented here, we have evaluated the repurposing of gliptins in a well-established PD animal model of DA depletion by characterizing the potential of Gosogliptin (PF-00734200) and Sitagliptin. *In vivo* (rat) studies were designed to evaluate gliptin-induced combined incretin receptor stimulation in a classical animal model of PD, the 6-hydroxydopamine (6-OHDA) unilateral medial forebrain bundle lesioned rat. This model was used consequent to the reproducible unilateral dorsal bundle lesion for striatal DA, differential DA release and receptor supersensitivity that results and underpins rotational behavior following methamphetamine challenge to support drug screening. Furthermore, the presence of the targets of the incretins, cells expressing the GLP-1R and GIPR, were evaluated in brain across age and in 6-OHDA challenged rodents as well as in human subjects with and without PD. Finally, immortalized cells and primary neuronal cell cultures were evaluated for neurotrophic/protective actions of single and combined GLP-1R and GIPR activation as a basis for further understanding actions of gliptin efficacy in our 6-OHDA rat studies.

**Materials and methods**

**Chemicals**

Sitagliptin was purchased from Beta Pharma Scientific (Branford, CT, USA). PF-00734,200 was a gift from Pfizer (Pfizer Inc., New York City, NY, USA). Routine chemicals were purchased from Sigma Aldrich (St Louis, MO, USA).

**Animals**

Mice, C57BL/6 males at 5, 15, 31-month-old (obtained from the NIA aging colony), were used to age-dependently evaluate the presence of the ultimate drug targets of DPP-4 inhibition, specifically GLP-1R and GIPR, in brain. Adult male 250 g Sprague-Dawley rats (Charles River Laboratories, 2 months old upon arrival) were used for PD-related in vivo studies. The use of animals was approved by the Animal Care and Use Committee, NIA and NIDA, NIH, IRP (Protocol #331-TGB-24 and #09-CNRB-9) and National Health Research Institutes, Taiwan (Protocol # 102068-A; 102102-A). Rats for the 6-OHDA study were single caged and were fed with 3 pieces of food chow (16.2 g or 5.4 g x 3) per animal per day, delivered at 11 am each day.

**Oral administration of Gliptins**

Gliptin treatment was initiated either prior to or after 6-OHDA lesioning (early and delayed treatment, respectively) as follows. (1) Early treatment: PF-00734,200 (2.5 mg), sitagliptin (2.5 mg), or vehicle was administered orally via diet admixture (within the 16.2 g chow) daily, starting from 7 days before to 35 days after unilateral 6-OHDA lesioning (Fig. 2A, and green line within Fig. 2D). An estimated dose for a 250-g rat under this treatment was 10 mg/kg/day. (2) Delayed treatment: PF-00734,200 (7.5 mg), sitagliptin (7.5 mg), or vehicle was administered orally via diet admixture (in 16.2 g chow) daily, starting from 7 days to 45 days after unilateral 6-OHDA lesioning (Fig. 3A). An estimated, clinically relevant, dose for a 250 g rat under this treatment was thus 30 mg/kg/day. Specifically for drug admixture into diet, drug was solubilized in 100% ETOH that was then added to the 16.2 g of food chow. Thereafter, the ETOH was evaporated off. The same procedure was followed for vehicle treated animals, but without either PF-00344,300 or sitagliptin. Animals were weighed twice weekly and the amount of drug added to the chow was modified accordingly to maintain the daily required dose. Chronic treatment with PF-00734,200 or sitagliptin did not alter body weight in 6-OHDA lesioned rats (Supplemental Fig 1). The drug doses evaluated in our rats (10 and 30 mg/kg daily, p.o.) were specifically chosen as they are equivalent to the human use of sitagliptin and PF-00734,200 following translation across animal species subsequent to normalization based on body surface area, in accord with FDA guidelines [33]. Specifically, the 10 mg/kg oral daily drug dose in our rat studies is equivalent to the routine human daily oral dose (100 mg in a 65 kg human) of these gliptins used in T2DM, and the 30 mg/kg rat dose is equivalent to a 3-fold higher dose that is well-tolerated in humans.

**Intracerebroventricular (i.c.v.) administration of PF-00734,200**

Following the exact same protocol described in Yin et al., 2012 [34] and Li et al., 2009 [35], anesthetized rats were injected i.c.v. with PF-00734,200 (15.4 nmole in 20 µL sterile physiological saline (n=6)) or vehicle (sterile physiological saline, 20 μL (n=8)) using a 25 μL Hamilton syringe at 10 min prior to 6-OHDA lesioning (Supplemental Fig 2). The coordinates for the i.c.v. injection were 0.8 mm posterior to bregma, 1.2 mm lateral (Left) to midline, and 3.7 mm ventral to the dural surface. The rate of infusion (4 μL/min) was adjusted by a microprocessor-controlled injector mounted to the stereotaxic frame (UMP4; World Precision Instruments, Sarasota, FL, USA). The needle was slowly removed after 2 min upon completion of the injection.

**Unilateral 6-OHDA lesioning**

Following the exact same protocol described in Yin et al., 2012 [34] and Harvey et al. 2004 [36], anesthetized rats were subjected to a 6-OHDA unilateral medial forebrain bundle lesion. Briefly, 6-OHDA (2.27 µg/µL x 5 µL in 0.9% NaCl containing 0.2 mg/ml ascorbic acid) was unilaterally injected into the medial forebrain bundle (-4.4 mm AP, 1.2 mm ML relative to bregma and 8.4 mm below skull) over 4 min through a syringe pump (Micro 4, WPI, Sarasota, FL), as approved under National Health Research Institutes, Taiwan, Protocol # 102068-A; 102102-A. Following this procedure, all animals were monitored daily for adverse signs of health for 1 week (in line with the approved protocols).

Our chosen study design was based on data derived from extensive prior animal studies that initially characterized the 6-OHDA unilateral medial forebrain bundle lesion PD model [37-40]. These studies evaluated animals with and without a lesion or drug treatment to provide baseline measures in relation to dopaminergic markers. These, as well as recent studies [41], demonstrated that dopaminergic markers are depleted in the dopaminergic rich areas of the striatum and SNc ‘ipsilateral’ to the 6-OHDA lesion, in comparison to the ‘contralateral’ side, and, importantly, contralateral side dopaminergic marker levels are similar to, and not statistically different from, animals without lesions. This indicates that there is no depletion of dopaminergic markers on the contralateral side of rats with a 6-OHDA unilateral lesion [37-40]. In the light of this, in the current study the dopaminergic markers quantified within the striatum and SNc ipsilateral to the 6-OHDA lesion were compared to those on the contralateral side within the very same animals. Furthermore, to evaluate drug actions, levels of dopaminergic markers in gliptin treated groups of 6-OHDA unilateral lesioned rats were compared to those of vehicle unilateral lesioned animals, in accord with other studies [42-44].

Our decision to use male rats in the present study was based on prior studies involving normal, ovariectomized as well as pubertal rodents that demonstrated that estrogens and possibly differences in neurotrophic factors provide significant neuroprotection to mitigate 6-OHDA damage in female rodents [42-44]. Consequently, to avoid potential confounds associated with estrogen generation in young female rodents, or a requirement of potentially ovariectomizing or using aging animals in a postmenopausal state, we performed our initial evaluation of gliptins in 6-OHDA lesion animal studies, reported herein, in young adult male rodents. We made this decision aware that gender differences in relation to the pharmacokinetics and tolerability of gliptins could potentially exist, and these could be evaluated in future studies in the event that a promising signal of efficacy is demonstrated in the investigation described herein.

**Methamphetamine-induced rotation**

The rotational behavior of rats was evaluated using a multichannel rotometer system (RotoMax, AccuScan Instruments, Inc, PA, USA), following the protocol of previous studies [45, 46]. For the early treatment study, methamphetamine (meth, 2.5 mg/kg, s.c.)–induced rotation was examined at 20 and 30 days after lesioning, as with prior studies [45, 46] (Fig. 2A). In the delayed treatment study, animals were challenged with meth (2.5 mg/kg, s.c.) 7 days after 6-OHDA lesioning as previously described [34]. Animals that rotated in excess of 300 turns/hour, indicative of a significant unilateral DA lesion, were selected for gliptins or vehicle treatment, and were randomized between groups (GraphPad Software, Boston, MA). Meth–induced rotation was re-examined at 20, 30 and 40 days after lesioning (Fig. 3A).

**Injection of BrdU**

BrdU (Sigma-Aldrich, St. Louis, MO) was injected parenterally (i.p.) from day 8 to day 20 after 6-OHDA lesioning at the dose of 50 mg/kg/day as a marker of neurogenesis. Animals were euthanized for immunostaining on day 21.

**Tyrosine hydroxylase (TH) and BrdU immunoreactivity**

BrdU and TH localization was determined by immunohistochemistry. Serial sections of the entire brain were cut at 30 μm thickness by cryostat. One series from every sixth section was stained for TH. To control for staining variability, specimens from all experimental groups were included in every batch and reacted together in a net well tray under the same conditions. Sections were rinsed in 0.1M phosphate buffer (PB), blocked with 4% bovine serum albumin (BSA) and 0.3% Triton x-100 in 0.1M PB. Sections were then incubated in a primary antibody solution mouse monoclonal anti-TH diluted in 4% BSA and 0.3% Triton x-100 in 0.1M PB, concentration 1:100 (Chemicon, Temecula, CA) and polyclonal anti-BrdU (1:500, Millipore, MA, USA) for 17-19 hr at 4°C. Sections were then rinsed in 0.1M PB and incubated in secondary antibodies for 1 hr, followed by incubation for 1 hr with avidin-biotin-horseradish peroxidase complex. Thereafter, sections were mounted on slides, and coverslipped. TH and BrdU immunoreactivity was examined under a fluorescent microscope. Sections were incubated without primary antibody as a control and observers were blinded as to treatment groups.

TH immunoreactivity and BrdU cell number in striatum were averaged from 3 brain sections with visible anterior commissure in each animal. TH immunoreactivity in SNc was measured every 6th section (30 μm per each section) throughout the extent of midbrain. A total of 7 sections from each animal were used for TH density analysis using ImageJ 1.52q software (National Institutes of Health, Bethesda, MD, USA). Nigral volume was analyzed using Cavalieri's method.

**Dopamine measurements by HPLC**

Brain samples were collected at 45 days after 6-OHDA lesioning. Striatal and SNc tissues were weighed and stored at −80°C until extraction. The tissues obtained from each animal were homogenized in 0.1 M perchloric acid and centrifuged at 13,000 g for 15 min. DA levels were measured by HPLC with electrochemical detection [47]. The analytical column was a Symmetry C18 3.5 µm (4.6×150.0 mm) from Waters (Milford, MA). The mobile phase consisted of 0.01 M sodium dihydrogen phosphate, 0.01 M citric acid, 2 mM sodium EDTA, 1 mM sodium octylsulfate, 10% methanol, pH 3.5 and was used at flow rate of 0.9 ml/min and a temperature of 25°C. The HPLC system consisted of an ESA automated injection system, an ESA 582 pump, and a Coulochem III detector (ESA Biosciences, Chelmsford, MA, USA). An EZChrom EliteTM chromatography data analysis system (ESA Biosciences) was used for data collection and analysis. DA content was calculated as nmole/g of tissue weight.

**Protein Extraction from tissues and Western blots**

Brain tissues were homogenized in T-PER Tissue Protein Extraction Reagent (Thermo Scientific, Waltham, MA, USA) in the presence of protease inhibitor (Halt Protease Inhibitor Cocktails from Thermo Scientific) using a Polytron homogenizer. For Western blotting, all samples, 50 µg total protein/lane was resolved by use of a NuPAGE Bis-Tris 10% precast gel (Invitrogen, Carlsbad, CA) and transferred onto a 0.2 mm PVDF membrane (Invitrogen). The blots were first blocked in 5% milk in TBST (tris buffered saline tween-20) at room temperature for 1 h, and then incubated in the same blocking solution containing primary antibodies overnight at 4°C (GLP-1R and GIPR antibodies were from Abcam (Cambridge, MA, USA) and used at a dilution of 1:500; Tyrosine hydroxylase monoclonal antibody was from Chemicon (Temecula, CA, USA) and used at a dilution of 1:2000; β-actin antibody was from Sigma and used at a dilution of 1: 2000; α-tubulin antibody from Santa Cruz Biotechnology (Dallas, TX, USA) was used at a dilution of 1: 5000). After sufficient washes with TBST, blots were incubated with appropriate HRP (horse radish peroxidase)-conjugated secondary antibodies for 1 h at room temperature. Blots were again washed in TBST and, thereafter, signals were detected by using SuperSignal West Pico or Femto Chemiluminescent Substrate (Thermo Scientific) according to the sensitivity requirement. Finally, blots were exposed to high-performance chemiluminescence film (GE Healthcare, Piscataway, NJ, USA) for an appropriate period of time, and densitometric quantification of the protein bands was performed by using a PC version of NIH IMAGE (ImageJ software). For striatum protein samples from the 6-OHDA PD rat model (Fig 1 B2,B3), blots were first incubated with primary antibodies against tyrosine hydroxylase and β-actin overnight at 4°C, then incubated for 90 min in goat anti-rabbit IR-700 nm, goat-anti-mouse IR-800 nm secondary antibodies (1:2,500, Li-Cor, Lincoln, NE, USA). The membranes were scanned using an Odyssey infrared imager (Li-Cor, Lincoln, NE, USA). Immunoblots were quantified with ImageJ.

**Active GLP-1 and GIP measurement in rat plasma and CSF**

Plasma was obtained from whole blood samples at the time of euthanasia (centrifugation 10,000xG 60 s at 4^o^C), and CSF was obtained from the cisterna magna. Samples were evaluated by ELISA using kits from IBL (Immuno-Biological Laboratories, Inc.) or from Millipore, following their protocols.

**DPP-4 activity**

Levels of DPP-4 activity in plasma and brain homogenate samples were quantified by use of a luminescent DPPIV-Glo™ Protease Assay (Promega, Madison, WI), using a 96-well plate format and following the manufacture’s protocol. For brain samples, final results were normalized to protein content.

**Neuroinflammatory measures in brain**

To quantify rat brain cytokine levels, brain tissues were homogenized in TPER (Tissue Protein Extraction Reagent) (ThermoFisher Scientific) using a Biomasher Homogenizer. Inhibitors of phosphatase and protease were added into the TPER to prevent protein degradation. Tissues were then sonicated for 10 pulses and centrifuged at 10,000g for 10 min at 4 °C. The supernatant was used to measure brain cytokine levels using rat TNF-α ELISA kits from e-Bioscience (San Diego, CA, USA) and rat IL-6 ELISA kits from Biolegend (San Diego, CA, USA). The final results were normalized to protein content, as measured by BCA (bicinchoninic acid) assay. Brain tissue Iba-1 and GFAP levels were quantified by Western blot (Iba-1 and GFAP antibodies Santa Cruz Biotechnology, Inc.) and used in 1:100 dilution).

**Rat pharmacokinetic study:** To define how accurately the selected sitagliptin and PF-00734,200 doses in our present rat study reflect those determined in prior human clinical studies, plasma and brain levels of sitagliptin and PF-00734,200 were evaluated in rats following a single oral dose of 10 mg/kg.

Specifically, sitagliptin or PF-00734,200 (10 mg/kg) was administered by oral gavage to adult male 250 g Sprague-Dawley rats (Charles River Laboratories, 2 months old upon arrival) under an approved Animal Care and Use Committee, NIA, NIH, IRP animal protocol (#331-TGB-24) (n=6). At 105 min thereafter, animals were euthanized. A blood sample was obtained and the plasma was separated (10,000 G, 60 s, 4^o^C) and frozen to -80^o^C. The vasculature was immediately cleared with physiological saline, and a section through the right cerebral hemisphere was obtained and flash frozen to -80^o^C. The 105 min sampling time was selected to provide approximately peak oral bioavailability [48]. The plasma and brain samples were later analyzed for sitagliptin or PF-00734,200 by LC-MS utilizing the methods of Zeng et al. [49] and Sharma et al., [50], respectively.

**Mouse brain tissue samples for GLP-1R/GIPR evaluation**

C57BL/6JN mice of 5, 15 and 31months of age (NIA aging colony, NIA Division of Aging Biology, Bethesda, MD) were euthanized (isoflurane followed by decapitation). Their brains were rapidly removed on wet ice, dissected, and immediately frozen and stored at -80^0^C.

**Human brain tissue samples for GLP-1R/GIPR evaluation**

Human PD and age-matched control brain samples (SNc) were generously provided by the Carroll A. Campbell Jr. Neuropathology Laboratory Brain Bank, Medical University S. Carolina, Charleston, SC.

**Neuronal cell culture studies**

Human SH-SY5Y neuroblastoma cells and rat VM neuronal cultures possess incretin receptors [35] and were prepared following the protocols of [34, 35]. Briefly, SH-SY5Y cells, obtained from American Type Culture Collection (ATCC, Manassa, VA), were sustained in a 1:1 mixture of Eagle's Minimum Essential Medium and Ham's F12 Medium supplemented with 10% heat-inactivated fetal bovine serum (FCS) and 100 U/mL penicillin/streptomycin (Invitrogen, Carlsbad, CA). Cells were kept at 37°C in a humidified incubator with 5% CO_2_ and 95% air. Medium was replaced every two days and the cells were split in a 1:3 ratio every 5 days (0.25% trypsin, 0.53 mM EDTA solution) or when they reached approximately 80% confluence.

Primary neuronal cultures were prepared from embryonic (E14-15) ventral mesencephalon (VM) tissue aseptically separated from timed-pregnant Sprague–Dawley rat fetuses (Charles River Laboratories, Wilmington, MA). VM tissue was then trypsinized (0.25%; Invitrogen, Carlsbad, CA) with gentle mixing (15 min, 37°C). After rinsing (pre-warmed DMEM/F-12 (Invitrogen)), cells were dissociated by trituration, counted and plated into 96-well (6.0 × 10^4^/well) cell culture plates pre-coated with poly-lysine (Sigma-Aldrich) and with culture medium (Dulbecco’s modified Eagle medium/F12 supplemented with 10% heat-inactivated fetal bovine serum, 1 mM L-glutamine and 2% B27 (Invitrogen)). VM cultures were then maintained (37°C, humidified atmosphere 5% CO_2_ and 95% air) and fed by exchanging 50% of media with feed media (Neurobasal medium (Invitrogen) with 2% B27 with antioxidants (+AO) supplement on DIV (days in vitro) 3 and 5). On DIV7, cultures were fed with feed media containing B27 supplement lacking antioxidants ((−) AO, Invitrogen).

For studies on neurotrophic actions, SH-SY5Y cells were treated with GLP-1, GIP or GLP-1+GIP (10 and 100 nM). Cell viability was measured by MTS assay at 48 hr (Promega, Madison, WI). For neuroprotection studies on VM primary cultures, freshly prepared 6-OHDA (100 μM in 20 μM ascorbic acid saline solution) or saline (with 20 μM ascorbic acid) was added to the wells on DIV 10, exactly 10 min following the addition of GLP-1, GIP, GLP-1+GIP or vehicle and with or without PF-00734,200 (1 nM). After incubation for 2 hr, cultures were washed with (−)AO B27 3 times. Incretin or vehicle was re-added to the wells during the last wash. Cells were returned to a 37°C incubator for 22 hr, and then fixed with 4% paraformaldehyde (PFA) for TH immunoreactivity evaluation. In relation to SH-SY5Y cells, parallel studies were undertaken in which cells were administered GLP-1, GIP or GLP-1+GIP (10 or 100 nM) or vehicle, and were either maintained in media lacking FCS for 48 hr (serum starvation) or challenged with 6-OHDA (30 μM) 2 hr later and then maintained for a further 24 hr. Likewise, cell viability was evaluated by MTS assay (Promega), rather than evaluation of TH levels, as TH expression can potentially be upregulated variably by phosphorylation following trophic factor treatment.

**Statistics**

To minimize the use of animals in the present study, useful outcome measures were selected from our prior studies for evaluation herein, and were supported by a power analysis. Values are expressed as means ± standard error of mean values throughout. Student’s t-test, and 1- and 2-way ANOVA tests were used for statistical analyses. ANOVA on ranks was used when the normality assumption was violated. Post-hoc Newman-Keuls test or Dunn’s test was used for all pairwise multiple comparisons. The Dunnett’s and Bonferroni correction were used, as required, for serial measurements. A statistically significant difference was defined as p < 0.05, and statistical values are noted in the Figures and text. No outliers or data was excluded.

**Results**

**Rodent and human brain expresses both GLP-1R and GIPR**

The brain receptor targets of the incretins, GLP-1 and GIP; specifically, GLP-1R and GIPR, were probed by Western blot and their protein expression levels were found to be preserved across age in mouse cerebral cortex (Fig. 1 A1, A2), were maintained in rat striatum following 6-OHDA lesioning (Fig. 1 B1), and also were confirmed present in human SNc in PD (Fig. 1 C1, C2)). Human SNc tissue was obtained from both Parkinson’s disease subjects vs. an age-matched control.

**Fig 1**. GLP-1R and GIPR were probed by Western blot and their protein expression levels were maintained across age in mouse cerebral cortex, were retained in striatum following unilateral 6-OHDA lesioning in rat, and also were preserved in human substantia nigra pars compacta in PD (all values are means ± SEM). (**A1**) GLP-1R and (**A2**) GIPR expression normalized to β-actin in mouse cerebral cortex was unchanged across age (5- to 31-months; p>0.05, n=3 per age group; one-way ANOVA; Bonferroni’s *post hoc* test). (**B1**) GLP-1R and GIPR expression levels normalized to β-actin in striatum were found to be similar in rats challenged with 6-OHDA unilateral medial forebrain bundle lesion on the left (L) side (p>0.05, n=3, Student T test between L and R striatum) in the presence of substantial left-sided dopaminergic cell depletion, as evaluated by tyrosine hydroxylase (TH) expression (**B2**) normalized to β-actin (**B3**). (**C1**) GLP-1R and (**C2**) GIPR expression together with α-tubulin in a single human control and two PD SNc brain samples (obtained from the Carroll A. Campbell Jr. Neuropathology Laboratory Brain Bank, Medical University S. Carolina, Charleston, SC).

**Early treatment with a low daily dose of gliptin reduced rotational behavior and protected against the loss of dopaminergic innervation in striatum after 6-OHDA lesioning in hemiparkinsonian rats**

Rats were treated with PF-00734,200, sitagliptin (both drugs 10 mg/kg daily, orally in 16.2 g of food), or vehicle starting from 7 days prior to unilateral 6-OHDA lesioning (day 0) and this was continued for a further 5 weeks. Animals were euthanized at 35 days after lesioning (as shown in Fig 2A). This drug dose (10 mg/kg daily, p.o.) in rat was specifically chosen as it is equivalent to the human use of sitagliptin and PF-00734,200 following translation across animal species subsequent to normalization based on body surface area, in accord with FDA guidelines [33]. Specifically, the 10 mg/kg oral daily drug dose in our rat studies is equivalent to the routine human daily oral dose (100 mg in a 65 kg human) of these gliptins used in T2DM [34].

In vehicle control animals, lesioning with 6-OHDA almost completely abolished tyrosine hydroxylase (TH) immunoreactivity within the ipsilateral striatum (Fig 2B). PF-00734,200 partially protected against the loss of TH activity in the lesioned striatum. A more prominent protective response was found in animals receiving sitagliptin (Fig 2B). TH pixel density in the striatum from 27 rats was averaged from 3 brain slices with visible anterior commissure in each rat (vehicle, n=10; PF-00734,200, p.o., n=9; sitagliptin p.o., n=8). In animals receiving vehicle, TH pixel density in the lesioned side striatum was reduced to 2.80 ± 1.04% of control (non-lesioned side striatum). Early oral treatment with sitagliptin or PF-00734,200 significantly increased TH pixel density in the lesioned striatum (Fig 2C, p<0.05; one way ANOVA on Rank + Dunn’s test).

Meth (2.5 mg/kg, s.c.)–induced rotational behavior was examined on days 20 and 30 following 6-OHDA, in line with prior studies [45, 46]. Using a two-way ANOVA, we determined that both gliptins, compared to vehicle, significantly reduced meth-mediated rotation in the unilaterally 6-OHDA-lesioned rats (F_2,51_=4.734, p=0.013; Fig 2D). Post-hoc Newman-Keuls analysis revealed that PF-00734,200 significantly reduced meth-mediated ipsilateral rotation (p=0.006). A small but significant difference was found between sitagliptin and vehicle groups (p=0.050). No difference was found between oral PF-00734,200 and sitagliptin groups.

In contrast to the neuroprotection induced by oral administration of gliptins, unilaterally 6-OHDA-lesioned rats that were treated with PF-00734,200 by the i.c.v. route were no different from similarly vehicle-treated animals, as evaluated for meth-mediated rotational behavior and TH immunohistochemistry (Supplemental Fig. 2).

**Fig 2**. Early treatment with low dose of gliptins reduced ipsilateral rotation and protected against the loss of dopaminergic innervation in striatum as well as substantia nigra pars compacta of hemiparkinsonian rats. PF‐00734,200 (10 mg/kg daily), sitagliptin (10 mg/kg daily), or vehicle was administered orally via diet admixture (in 16.2 g chow) daily, starting from 7 days before unilateral 6‐OHDA lesioning (green line within (**A** and **D**)). Daily drug/vehicle dosing was continued and methampphetamine-mediated rotation was quantified at 20 and 30 days post-lesion. Animals were euthanized, thereafter, and TH immunoactivity examined in striatum and SNc (all values are means ± SEM).

Striatum: (**A**) Time line of early treatment protocol. (**B**) Representative photomicrographs from three separate animals per group (i.e. oral PF‐00734,200 (PF), sitagliptin (Sita), vehicle (Veh). (**C**) TH immunoreactivity was averaged from three coronal sections at the level with visible anterior commissure from each animal. TH levels in the lesioned side striatum were normalized to the corresponding non‐lesioned side striatum on the same brain slide. Whereas the TH level in animals receiving vehicle was almost completely abolished, early treatment with sitagliptin or PF‐00734,200 significantly preserved TH immunoactivity in the lesioned striatum (*p<0.05, one way ANOVA on Rank + Dunn’s test). (**D**) Rotation was induced by administration of 2.5 mg/kg methamphetamine at days 20 and 30 after lesioning. PF‐00734,200 significantly reduced rotation (*p=0.006), and a smaller but significant difference was found between sitagliptin and vehicle groups (*p=0.050).

Substantia nigra: (**E**) TH activity was almost completely abolished after unilaterally 6‐OHDA lesioning, as evident in the vehicle (Veh) group (Scale bar = 1mm for **E**). Oral administration PF or Sita (both 10 mg/kg daily) antagonized 6‐OHDA-mediated loss of TH activity (**F**). The labels within (**F**), i.e., A1,A2,B1,B2,D1,D2,E1 and E2, in each high magnification panel - correspond to the blocks in (**E**). As evident in (**F**), oral treatment with PF or Sita partially ameliorated 6‐OHDA induced loss of TH cells and fibers in SNc (within (**F**), PF: D1, D2 and Sita E1 and E2). Calibration bar = 200 µm. (**G** and **H**) TH immunoreactivity was quantified on 7 sections from each animal. The distance between sections was 240 µm. (**G**) In the lesioned side SNc, treatment with Sita or PF significantly increased TH activity (#p<0.001, compared to Veh control). (**H**) In the non‐lesioned side SNc, PF or Sita did not alter the TH immunoreactivity (Veh n=10; PF n=9; Sita n=8).

**Early treatment with gliptins reduced dopaminergic degeneration in the lesioned substantia nigra**

The protective response of gliptins was also found in SN (Fig 2E). Almost no TH (+) cells were found in the lesioned SNc in vehicle treated animals. In contrast, TH immunoreactivity in the lesioned SNc was increased in comparison to the vehicle-treated group following treatment with PF-00734,200 or sitagliptin. At high magnification, TH (+) cells and fibers were found to be partially preserved in the lesioned SNc (Fig 2F**)**. TH immunoreactivity was further quantified in the all animals. A total of 7 brain sections from each animal were used. The distance between slices was 240 µm. We found that in the non-lesioned side SNc, early treatment with PF-00734,200 or sitagliptin did not alter the TH immunoreactivity (Fig 2H). In the lesioned side SNc, TH immunoactivity was significantly reduced in animals receiving vehicle (Fig 2G). Treatment with gliptins significantly altered TH immunoactivity (p<0.001, F_2,175_=21.230, two-way ANOVA). Post hoc Newman-Keuls test indicated that sitagliptin or PF-00734,200 significantly increased TH activity (p<0.001, Fig 2G).

**Delayed oral treatment with a higher dose of gliptins attenuated rotational behavior in 6-OHDA lesioned rats**

Forty-six rats received unilateral 6-OHDA lesioning on day 0 and were fed with regular food (16.2 g per day) for 6 days. Meth-induced rotational behavior was examined on day 7 after 6-OHDA lesioning (Fig. 3A). On the basis of this, animals were randomly separated into 3 groups to equalize rotational behavior and were fed with food (16.2 g per day) containing a high dose of gliptins (30 mg/kg/day) or vehicle from days 7 to 45. There was no difference in the rotation prior to drug treatment (i.e., on day 7) among these three groups (p=0.383, One way ANOVA). Averaged rotation on day 7 was 526.7 ± 41.6 turns/60 min. The 30 mg/kg gliptin daily dose was selected as it is equivalent to a 3-fold greater than routine human dose of sitagliptin and PF000734,200 that is well tolerated [34], calculated in accord with FDA guidelines [33].

Meth-induced rotation behavior was examined again on days 20, 30 and 40 after lesioning. Delayed oral treatment with gliptins significantly reduced rotation (Fig 3B, p=0.008, F_2,122_=5.078, Two-way ANOVA). Posthoc Newman-Keuls analysis indicates that sitagliptin (p=0.014) or PF-00734,200 (p=0.005) significantly attenuated rotational behavior as compared to vehicle. In another set of animals (n=14), a low dose of PF-00734,200 (10 mg/kg daily) or vehicle was given daily from day 7 to day 30. PF-00734,200, and this dose did not alter rotational behavior in hemi-parkinsonian rats (Supplemental Fig 3).

**Fig 3.** Delayed oral treatment with PF‐00734,200 (PF) or Sitagliptin (Sita) ameliorated meth‐induced ipsilateral rotation in unilateral 6‐OHDA induced hemiparkinsonian rats. (**A**) High dose PF or Sita (30 mg/kg daily, p.o.) or vehicle was administered orally to animals, initiated 7 days after a unilateral 6‐OHDA lesion. Animals were injected with meth (2.5 mg/kg, s.c.) 7 days after 6-OHDA lesioning to randomly distribute animals that rotated in excess of 300 turns/hour (indicative of a significant unilateral DA lesion) across treatment groups, and meth–induced rotation was thereafter re-examined at 20, 30 and 40 days post lesioning. (**B**) A significant reduction in rotation was noted in animals receiving PF or Sita (all values are means ± SEM; *p=0.009, two‐way ANOVA + Newman‐Keuls test), as compared to the vehicle controls (Veh n=20; PF n=14; Sita n=12).

**TH and BrdU immunoreactivity in 6-OHDA unilateral lesion-challenged rats receiving delayed oral treatment with gliptins**

Sixteen rats receiving delayed treatment were given daily 5′-bromo-2′-deoxyuridine (BrdU 50 mg/kg i.p.: an established marker of cell proliferation/neurogenesis) injection from day 8 to day 20. Brain tissues were collected on day 21 for TH and BrdU immunoreactivity. Unilateral 6-OHDA almost completely abolished TH immunoreactivity in the ipsilateral striatum in vehicle animals (Fig 4). Gliptins partially protected against the loss of TH immunoactivity in striatum **(**Fig 4A). BrdU-labeled cells were found mainly in the subventricular zone (SVZ) and striatum (Figs 4A and B). Delayed treatment with PF‐00734,200 or sitagliptin enhanced BrdU labeling in the lesioned striatum, but not in the SVZ (Fig 4B).

TH pixel density was averaged from 3 brain sections with visible anterior commissure in each animal. Sitagliptin significantly increased TH pixel density in the lesioned striatum (Fig 4C (C1), p=0.041, F_2,13_=4.121, one way ANOVA; p=0.036, post hoc Newman-Keuls test). There is a trend, but not a statistically significant PF‐00734,200-mediated increase in TH immunoactivity. The number of BrdU labeled cells in SVZ or striatum (Fig 4C (C2 and C3, respectively) )was quantified and averaged in the brain sections with visible anterior commissure. Sitagliptin or PF-00734,200 did not alter BrdU labeling in the SVZ (Fig 4C (C2), p= 0.236); however, both drugs significantly increased BrdU labeling in the striatum (Fig 4C (C3), p= 0.007, F=7.436, One-way ANOVA; p=0.012 and 0.006, posthoc Newman Keuls test).

**Fig 4.** Delayed gliptin treatment enhanced TH immunoreactivity and BrdU labelling in lesioned striatum in unilateral 6‐OHDA induced hemiparkinsonian rats. Either Sitagliptin (Sita) or PF‐00734,200 (PF) (both 30 mg/kg p.o. daily) or vehicle (Veh) was administered from day 7 onwards following unilateral 6‐OHDA‐ lesioning. BrdU (50 mg/kg i.p. daily) was administered from day 8 post‐lesion to day 20, and animals were euthanized on day 21 for collection of brain tissues for evaluation of TH and BrdU immunoreactivity (all values are means ± SEM). (**A**) Sita (A2) or PF (A3) partially protected TH activity in the lesioned striatum as compared to vehicle-treated animals (Scale bar = 200 μm). BrdU immunoreactivity was found to be chiefly present within the subventricular zone (SVZ) in vehicle treated animals. Delayed treatment with gliptins did not appears to enhance BrdU labeling bilaterally in the SVZ (Veh n=5; PF n=6; Sita n=5). (**B**) BrdU‐labeled cells were found predominantly in the SVZ (left panel) but also in the striatum (right panel) (Scale bar = 100 μm). Whereas treatment with PF or Sita did not enhance BrdU immunoreactivity in the SVZ (B2 and B3, as compared to B1 (left panel)), an increase in BrdU labeling was found in lesioned striatum after PF or Sita treatment (B2 and B3, as compared to B1 (right panel)), (Veh n=5; PF n=6; Sita n=5). (**C**) TH (C1) and BrdU (C2 and C3) immunoreactivity was averaged from three coronal sections at the level with visible anterior commissure from each animal. (C1) TH immunoactivity in the lesioned side (L: left) striatum was normalized to the corresponding non‐lesioned side (R: right) striatum on the same brain slide. Delayed treatment with Sita (30 mg/kg daily, p.o.) significantly increased TH activity in the lesioned striatum (*p=0.037, one-way ANOVA + Newman‐Keuls test). (C2 and C3) BrdU immunoreactivity in the animals treated with gliptins was normalized to the mean BrdU immunoactivity in animals treated with vehicle (Veh). (C2) No difference was found in the SVZ (p=0.236). (C3) Post treatment with PF-00734,200 (PF) or Sita (both 30 mg/kg daily, p.o.) significantly increased BrdU labeling in the lesioned striatum (*p=0.007, One-way ANOVA). (Veh n=5; PF n=6; Sita n=5).

This protective action after delayed gliptin treatment was also found in SNc. Almost no TH immunoreactivity was found within the lesioned side SNc in animals receiving vehicle-treated chow. Delayed treatment with gliptins partially ameliorated the loss of TH cell density in this area (Fig 5 A&B). TH immunoreactivity was analyzed in every 6^th^ section through the extent of the midbrain. A total of 7 brain sections from each animal was used for TH density analysis. SNc volume was analyzed using Cavalieri's method. In the non-lesioned side SNc, TH cell density was not altered by gliptin treatment (p=0.415, One-way ANOVA, Fig 5C). 6-OHDA lesioning significantly reduced TH cell density in all animals. TH cell density was reduced to 1-2 % in animals receiving vehicle (Fig 5D). Sitagliptin or PF-00734,200 significantly increased TH cell density in the lesioned SNc (Fig 5D, p<0.05, ANOVA on Rank + Dunn’s test).

**Fig 5.** Delayed oral gliptin treatment provides partial protection against 6‐OHDA-mediated neurodegeneration in the SNc dopaminergic neurons (all values are means ± SEM). (**A**) Photomicrographs represent typical TH immunoreactivity in the ventromesencephalic region of 3 animals at day 21 post lesioning. Almost no TH immunoactivity was found in the lesioned SNc area of a rat receiving vehicle (Veh). Delayed treatment with sitagliptin (Sita) or PF‐00734,200 (PF) (both 30 mg/kg daily, p.o.) partially mitigated the loss of TH immunostaining within the lesioned side of the SN. (**B**) At higher magnification, treatment with Sita and PF increased the TH immunoreactivity in the lesioned SNc (D2 vs. B2; C2 vs. B2). (Scale bars. A: 100 µm, B: 18 µm). (**C**) Gliptins did not alter cell density in the non‐lesioned side SNc (p=0.415). (**D**) Delayed treatment with PF or Sita significantly increased TH cell density in the lesioned side SNc (p=0.007). Scale bar = 100 μm. (Veh n=5; PF n=6; Sita n=5).

**Dopamine levels in striatum and substantia nigra**

A total of 49 rats received unilateral 6-OHDA lesioning on day 0 and were given oral PF-00734,200 (30 mg/kg daily, n=18), sitagliptin (30 mg/kg daily, n=14), or vehicle (n=17) from day 7 to day 45. Another 8 non-lesioned rats were fed with regular diet (16.2 g per day) without gliptins and were used as naïve controls. SNc and striatal tissues were collected for DA analysis by HPLC on day 45. Tissue DA concentrations in the non-lesioned side striatum or SNc of 6-OHDA lesioned rats were not statistically different from those of naive controls (data not shown). DA levels in the lesioned side (left or L) striatum and SNc were thus normalized to the non-lesioned side (right or R) from each animal and were expressed as L/R ratio (Fig 6). Administration of 6-OHDA reduced the DA L/R ratio to 0.037 (i.e., 3.7 ± 0.9 %) in the striatum and to 0.214 (i.e., 21.4 ±3.2 %) in SNc in vehicle animals (Fig 6). Delayed oral treatment with sitagliptin significantly increased DA levels in the lesion side striatum (Fig 6A) and SNc (Fig 6B, p<0.05, One-way ANOVA on Rank + Dunn’s test). A significant increase in DA levels in SNc was also found in the 6-OHDA-lesioned animals receiving sitagliptin, as compared to PF-00734,200 (p<0.05, One way ANOVA on Rank + Dunn’s test; Fig 6B). In animals receiving PF-00734,200 (10 mg/kg daily from days 7 to 45, orally, n=8), DA levels in SNc or striatum were not significantly altered (see Supplemental Fig 3).

**Fig 6**. Delayed treatment with high dose sitagliptin (Sita) mitigates dopamine (DA) loss on the side of the lesion in unilateral 6‐OHDA induced hemiparkinsonian rats. Unilaterally 6‐OHDA lesioned rats were given PF-00734,200 (PF) (30 mg/kg daily), Sita (30 mg/kg daily), or vehicle (Veh) orally from day 7 to day 45. Striatal and SNc tissue samples were collected on day 45 post‐lesion for HPLC analysis. All data in the lesioned side (left or L) striatum and SNc were normalized to the non‐lesioned side (right or R) from each animal and were expressed as L/R ratios. Delayed treatment with Sita significantly increased DA levels in the lesion side (**A**) striatum and (**B**) SNc. All values are means ± SEM; *p<0.05, One-way ANOVA on Rank + Dunn’s test. Veh n=17; PF n=18; Sita n=14

**DPP-4 inhibition, incretin levels and markers of inflammation**

Plasma, brain (striatum) and CSF samples were obtained from a subset of rats that received unilateral 6-OHDA lesioning on day 0 and were given oral PF-00734,200 (30 mg/kg daily), sitagliptin (30 mg/kg daily), or vehicle from day 7 to day 45 in relation to brain DA level evaluation (Fig. 6, above), to support quantification of DPP-4 activity, incretin levels and markers of inflammation. Plasma DPP-4 activity was significantly inhibited in excess of 60% by sitagliptin and PF-00734,200, and brain activity by in excess of 20% (Fig 7A, p<0.001 plasma, p<0.01 brain, ANOVA on Rank + Dunn’s test). This resulted in elevated levels of endogenous incretins in plasma and CSF (Fig. 7B and C), and a decline in brain markers associated with neuroinflammation (evaluated by TNF-α, IL6, Iba-1 and GFAP protein levels (Fig. 7D)). This decline in brain inflammatory markers is particularly relevant in the light of the known presence of neuroinflammation in human PD [3].

**Fig 7**. Delayed oral gliptin treatment induces reductions in plasma and brain DPP-4 activity, a rise in plasma and CSF incretin levels and a reduction in neuroinflammatory markers in unilateral 6‐OHDA induced hemiparkinsonian rats. Unilaterally 6‐OHDA lesioned rats were given PF-00734,200 (PF) (30 mg/kg daily), Sita (30 mg/kg daily), or vehicle (Veh) orally from day 7 to day 45. Plasma, CSF and brain tissue samples (lesioned side) were collected on day 45 post‐lesion (all values are means ± SEM). Delayed treatment with Sita and PF significantly inhibited (**A**) DPP-4 activity in plasma and brain (top and bottom, respectively, in striatum), elevated incretin levels in (**B**) plasma and (**C**) CSF (GIP (top), GLP-1 (bottom)), and reduced levels of the inflammatory markers TNF-α, IL-6, Iba1 and GFAP (**D**) on the lesioned side of brain (striatum). *p<0.05, *p<0.01, *p<0.001, One-way ANOVA, Dunnett’s multiple comparison test vs. Veh group (n shown within Figure). The small volume of CSF available for sampling from the cisterna magna proved to be restrictive in gaining data regarding sitagliptin action on GLP-1 levels in Fig. 7C.

**Plasma and brain levels of sitagliptin and PF-00734,200 in:** To verify that the selected sitagliptin and PF-00734,200 doses evaluated in our rat 6-OHDA unilateral lesion study are equivalent to those achievable in humans, plasma and brain levels of both gliptins were quantified in rat following a 10 mg/kg oral dose, and are shown in Table 1. Total and free sitagliptin concentrations in rat plasma were substantially greater than respective levels in brain, and demonstrate restricted brain entry of sitagliptin following oral administration. In contrast, more substantial concentration of PF-00734,200 were found in rat brain (brain plasma ratio 0.62).

|  | **Plasma (Pl)** |  | **Brain (Br)** |  | **Ratio** |
| --- | --- | --- | --- | --- | --- |
| **Compound** | Pl_Total_ nmol/L | Pl_Free_ nmol/L | Br_Total_ nmol/L | Br_Free_ nmol/L | Br/Pl ratio  (Total) |
| Sitagliptin | 976 ± 145.8 | 868 ± 129.7 | 71 ± 13.4 | 13 ± 2.4 | 0.07 |
| PF-00734,200 | 922 ± 205.7 | 830 ± 185.2 | 574 ± 102.9 | 195 ± 34.9 | 0.62 |

**Table 1.** Plasma (Pl) and brain (Br) total and free concentrations of sitagliptin and PF-00734,200 in rat at 105 min (the predicted plasma peak (C_max_) following oral 10 mg/kg administration) (n = 5 to 6 animals per group, mean value in nmol/L ± SEM).

**GLP-1 and GIP receptor activation provides neurotrophic/protective actions in neuronal cultures**

Our hypothesis is that elevated levels of the endogenous incretins GLP-1 and GIP that result from the use of a DPP-4 inhibitor provide neurotrophic/protective actions in addition to the anti-inflammatory effects described above. Both our research [51] and the prior work of others [52] have demonstrated the neurotrophic/protective actions of single as well a combined GLP-1 and GIP receptor activation. To confirm this, we treated SH-SY5Y cells (a human immortal neuronal cell line with dopaminergic markers [53]) with GLP-1, GIP, GLP-1+GIP or vehicle in the absence and/or presence of a neurotoxic challenge with either 6-OHDA (30 μM) or serum starvation. Pilot studies were undertaken to determine ‘subtherapeutic’ concentrations of single GLP-1 and GIP treatment that were then used to evaluate neurotrophic/protective actions of the combined GLP-1+GIP treatment. As shown in Figure 8A, the combination of subtherapeutic doses of single GLP-1 or GIP receptor agonism resulted in a neurotrophic action for GLP-1+GIP (p<0.01). Similarly, the combination of subtherapeutic doses of GLP-1 and GIP alone (Fig. 8B) provided neuroprotection against both 6-OHDA (p<0.05) and serum starvation (p<0.05) challenges. These results indicate that the joint action of ordinarily subtherapeutic concentrations of each incretin provide neurotrophic/protective benefit when combined.

To confirm that such benefit translates to primary neurons, rat VM cultures were challenged with a toxic concentration of 6-OHDA (100 μM) and rescued by the addition of GLP-1+GIP, particularly in the presence of the DPP-4 inhibitor PF-00734,200 (1 nM) (Fig. 8C).

**
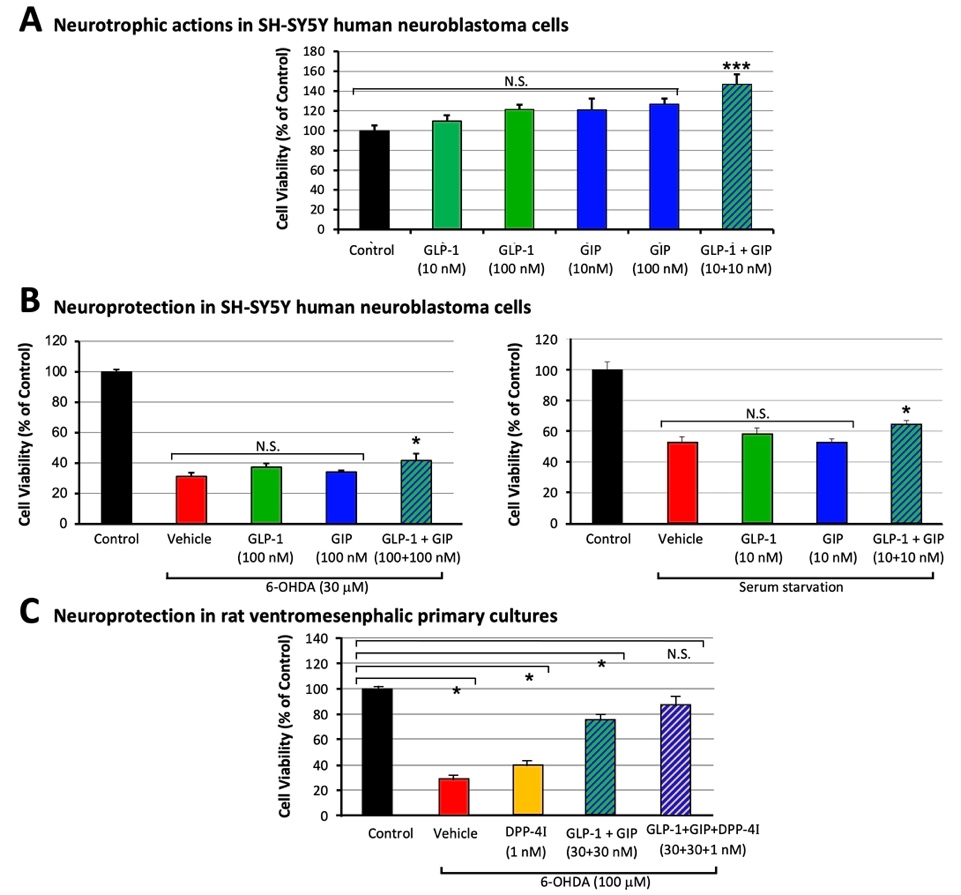
**

**Fig 8**. Incretins GLP-1 and GIP, and particularly when combined, possess neurotrophic and neuroprotective actions in neuronal cell cultures. SH-SY5Y cells were treated with single and combined concentrations of GLP-1 and GIP in the absence (**A**) and presence of cellular challenge (**B**). (**A**) The combination of subtherapeutic concentrations of GLP-1 and GIP, alone, resulted in neurotrophic action (i.e., an increase in cell viability vs. the Control group) and (**B**) significantly ameliorated 6-OHDA and serum starvation induced loss of cellular viability (vs. the Vehicle challenge group). (**C**) GLP-1+GIP mitigated 6-OHDA mediated cell death in rat primary VM neurons, particularly in combination with DPP-4 inhibition (DPP-4I: PF-00734,200 1 nM). All values are means ± SEM; *p<0.05, *p<0.001, N.S.: no significant difference, one-way ANOVA, Dunnett’s multiple comparison test vs. Vehicle or Control group (n = 3 to 4 per group).

**Discussion**

Dipeptidyl peptidase-4 is a cell surface serine peptidase that cleaves N-terminal dipeptides possessing the second residue of a proline or alanine, such as in the incretins GLP-1 and GIP [7-9, 32, 54]. In addition to its localization to cellular membranes, a soluble DPP-4 (sDPP-4) form abundantly occurs in plasma and biological fluids that retains peptidase activity but has no tethering transmembrane or cytosolic domains [54]. DPP-4 protein is highly expressed within the small intestine, kidney and lungs [54, 55]. In relation to the nervous system, high DPP-4 expression has been described in the developing brain and spinal cord, but a barely detectable level of protein is found in adult healthy brain [56]. In several studies, DPP-4 distribution has been analyzed by autoradiography after intravenous administration of the labeled DPP-4 ligand BI1356 [56], and available data suggest that DPP-4 is mainly a peripheral enzyme. Nevertheless, DPP-4 immunoreactivity has been reported in striatum, on the microvasculature endothelial wall of central and peripheral nervous systems [57], and on the luminal surface of ependymal cells in the cerebral ventricles [58]. Notably, whereas DPP-4 immunoreactivity is generally not found in naïve adult brain, its expression is time-dependently seen in microglia, astrocytes and neurons following cerebral ischemia [59]. In spinal cord, inflammation caused a 5-fold elevation in DPP-4 protein levels, particularly in astrocytes, via post-transcriptional regulatory mechanisms following ischemia, and DPP-4 inhibition provided anti-hyperalgesic effects [60]. The regional localization and levels of DPP-4 and sDPP-4, particularly in an inflammatory or pathological state, can potentially lower circulating endogenous incretin levels and, in particular, those that gain access into the central and peripheral nervous system.

Our hypothesis is that elevated incretin levels in brain and plasma, induced by gliptin-mediated inhibition of DPP-4 activity, will provide both neuroprotective and neuroregenerative actions in cell culture and *in vivo* following lethal and sub-lethal insults to dopaminergic neurons. This hypothesis is supported by preclinical studies of incretin mimetics across cellular and animal models of dopaminergic cell loss and PD [35, 61, 62] and, additionally, by the successful efficacy of the GLP-1 receptor agonist Exenatide in patients with moderate PD [26-30]. Interestingly, in human epidemiological studies, patients with T2DM are at a 35% elevated risk for developing PD [63]. However, this is significantly reduced in those patients taking incretin mimetics or DPP-4 inhibitors [64, 65] in line with our administration of gliptins prior to 6-OHDA unilateral lesion mitigating ensuing PD-associated effects in our pretreatment animal model. Our prior studies [51, 66, 67] as well as those of others [18, 23, 25, 68] have, in particular, demonstrated that unimolecular dual (GLP-1+GIP) receptor agonist incretin mimetics, as well as triple (GLP-1+GIP+ Glucagon) receptor agonists, are superior to single GLP-1 receptor agonists in providing neuroprotective and neurotrophic actions in cellular and animal models of neurodegenerative disorders [19, 22]. This is confirmed in our SH-SY5Y and VM cellular studies herein (Fig. 8). In these studies, the co-administration of a subtherapeutic concentration of GLP-1 or GIP singly, resulted in a statistically significant neurotrophic (Fig. 8A) or neuroprotective (Fig. 8B) action when GLP-1 and GIP were combined. Notably, DPP-4 inhibition simulates dual agonist incretin mimetics and, unlike subcutaneous injection of a therapeutic peptide, gliptins are small molecular weight, inexpensive, orally bioavailable drugs. Our *in vivo* studies in rodents support the presence of both drug targets, the receptors for GIP and GLP-1, in brain across age, reported here for the first time, and additionally demonstrated the preservation of these receptors in striatum following a 6-OHDA unilateral medial forebrain bundle lesion as well as in human PD brain (Fig. 1). Future follow up studies in a larger number of samples would be valuable.

Optimization of the 6-OHDA model in the rat was undertaken to support neuroprotection and neuroregeneration efficacy studies, in order to evaluate the therapeutic value of gliptins under steady-state conditions that mirror their clinical use in humans in relation to both route of administration (oral) and dose – following normalization between species based on body surface area, in accord with FDA guidelines [33]. Again, our choice of gliptin doses is based on translation of the routine human dose in T2DM (100 mg in a 65 kg patient: equivalent to 10 mg/kg in a rat) and a three-fold higher tolerated dose in humans [34] (equivalent to 30 mg/kg in a rat). Quantification of plasma sitagliptin and PF-00734,200 was undertaken in rat following a 10 mg/kg oral dose (Table 1) and demonstrated drug levels that closely align with those reported in human studies following administration of the routine dose used for T2DM [69, 70]. Specifically, a total plasma level of 976 nmol/L sitagliptin was found in our rat study and concentrations of 959 nmol/L [70], 817 nmol/L [69] , and 747 nmol/L [71] have been reported in human plasma samples. Hence, the present rat study truly evaluated gliptin drug doses supporting the repositioning sitagliptin and PF-00734,200 for PD, rather than potentially clinically “irrelevant” doses that are not achievable in humans. In this light, evaluation of neuroprotective actions was undertaken by initiating gliptin treatment in rat prior to a 6-OHDA unilateral lesion. Both PF-00734,200 and sitagliptin (10 mg/kg/day) significantly reduced meth-induced rotational behavior and mitigated depletion of TH immunoreactivity both within the striatum and SNc (Fig. 2). Evaluation of neuroregenerative actions was undertaken by initiating gliptin treatment 7 days post 6-OHDA unilateral lesion, a time associated with a marked loss of dopaminergic neuronal phenotypic markers. Gliptin treatment (sitagliptin or PF-00734,200 – both at 30 mg/kg daily) lessened meth-induced rotational behavior (Fig. 3). This was associated with a gliptin-induced mitigation in 6-OHDA induced loss of brain DA levels, particularly within the SNc (Fig. 6).

Analysis of TH immunohistochemistry demonstrated a gliptin-induced partial protection of the SNc (Fig. 5), in accord with elevations of DA within this brain region. Specifically using immunohistochemical analysis, we found that TH immunoreactivity in striatum was significantly protected by early oral treatment with gliptins (Fig. 4). In contrast, no protection was found in animals receiving i.c.v. gliptin (PF-00734,200) (Supplemental Fig. 2). In this scenario, the direct administration of a gliptin into the brain bypasses its normal systemic actions on incretins that are released from gastrointestinal L and K cells. This suggests that the neurotrophic/protective efficacy afforded by systemic (oral) gliptin administration was not mediated by a direct brain gliptin neuroprotective effect but, rather, by the systemic gliptin action to inhibit DPP-4 and, by this means, to elevate CNS incretin levels secondary to elevation of systemic levels. Whereas systemic GLP-1 and GIP are known to enter the brain [14-19], sitagliptin’s brain access is remarkably low (brain/plasma ratio 0.07 (Table 1) and PF-00734,200’s more substantial (brain plasma ratio 0.62). Likewise, we found that these gliptins reduced dopaminergic neurodegeneration in the SNc region. In animals receiving delayed gliptin treatment, a significant increase in TH immunoreactivity in striatum or SNc was found after chronic sitagliptin treatment. In this regard, PF‐00734,200 was less potent than sitagliptin in rescuing dopaminergic neurons in SNc (Fig. 4). In relation to levels of DPP-4 inhibition and lowering markers of neuroinflammation, both gliptins demonstrated largely similar efficacy (Fig. 7). Potentially accounting for sitagliptin’s low brain concentration and brain/plasma ratio following oral administration (Table 1), this drug has been reported to be a substrate for the multi-drug resistance transporter P-glycoprotein at the level of the kidney [72]. This same drug efflux transporter is present at the level of the cerebral microvasculature and likely accounts for sitagliptin’s low brain levels, which are 8- to 10-fold greater in mutant mice that lack a functional P-glycoprotein transporter [72]. The mitigation of markers of dopaminergic loss (TH immunoreactivity, DA, etc.) following a unilateral 6-OHDA lesion in the presence of a very low sitagliptin brain concentration provides further support of the hypothesis that sitagliptin’s brain efficacy is mediated in large part by induced elevations in GLP-1 and GIP, rather than by direct drug brain levels.

The protective effect of oral gliptins was further supported by tissue DA HPLC analysis at 1-2 months after lesioning. Specifically, lesioning with 6-OHDA greatly reduced DA levels in SNc and striatum. Similar to the TH immunostaining, loss of DA levels on the lesion side striatum and SNc was significantly mitigated by delayed treatment with sitagliptin (Fig. 6). We did not find a significant amelioration in DA loss by PF‐00734,200. These biochemical data suggest that sitagliptin is more potent than PF-00734,200 in protecting against 6-OHDA–mediated DA loss in striatum or SNc.

Our in vivo gliptin studies used the unilateral 6-OHDA lesion model of PD of Ungerstedt and colleagues [37-40] in which the stereotaxic unilateral injection of 6-OHDA into the medial forebrain bundle of rats results in a marked loss of classical dopaminergic markers and DA levels both in the SNc due to retrograde axonal transport as well as in dopamine terminals in the striatum ipsilateral to injection. In this model, pharmacological treatment with amphetamine (or an analogue) that causes DA release and inhibition of uptake, results in a rotational behavior consequent to a disparity of synaptic DA levels in the lesioned vs. unlesioned side [37, 73, 74]. Under ordinary circumstances, in the absence of pharmacological challenge, lesioned animals largely behave relatively normally. Additionally, sham (unlesioned) animals show no difference in their dopaminergic innervation or DA levels on either side of the brain and, hence, have no turning response to amphetamine challenge [38]. In this respect, the non-lesioned side of the brain is widely used as a ‘control’ in relation to the lesioned side when quantifying dopaminergic markers in the unilateral 6-OHDA lesion model (Figure 3 in [41]). Furthermore, the vehicle-treated unilateral 6-OHDA lesioned group is used as a ‘control’ when evaluating the actions of an experimental drug to mitigate dopaminergic marker loss [42-44].

Our results are in line with prior preclinical reports demonstrating that sitagliptin as well as the GLP-1R agonist liraglutide mitigated motor deficits, striatal DA loss, SNc dopaminergic neuronal degeneration and neuroinflammation in a rat rotenone model of PD [75, 76]. Vildagliptin, similarly, has been reported to mitigate striatal DA loss, neuroinflammation and motor deficits in the rat rotenone PD model [77]. It was also reported to reduce neuronal degeneration and markers of oxidative stress, and to normalize neurotransmitter and neurotrophic factor levels in the striatum of a rat 3-nitropropionic acid (3NP) model of late-stage Huntington’s disease [78]. Likewise, administration of saxagliptin in the rat rotenone PD model has been described to preserve TH levels within the SNc and mitigate striatal declines in DA and TH levels, as well as to lower markers of neuroinflammation and apoptosis [79]. In a similar manner, alogliptin has been recently reported to do the same in rotenone challenged PD rats [80]. As recently reviewed by Maanvi and colleagues [81], gliptins have additionally demonstrated neuroprotective and anti-inflammatory properties sufficient to mitigate behavioral deficits in selected rodent models of AD and ischemic stroke, thereby supporting their therapeutic potential across different models of neurodegeneration.

In the light of two prior reports demonstrating that linagliptin can augment neural stem cell proliferation after ischemic stroke in diabetic mice [82] and that sitagliptin augmented hippocampal neurogenesis in high-fat-fed mice [83], we evaluated neurogenesis in our 6-OHDA PD model. This was undertaken by post-lesion administration of BrdU and assessment of its immunoreactivity at the level of the SVZ, striatum and SNc. Both gliptins augmented neural progenitor cells bilaterally as assessed 21 days post lesion. BrdU has a short half-life of 2 hours and can label newly proliferating cells on the days of BrdU injection. We and others have previously demonstrated that brain injury increases BrdU labeled cells chiefly within the SVZ in an animal model of stroke [84, 85]. These BrdU-labeled cells also co-expressed neural progenitor cell (NPC) markers, such as Musashi-1, in the SVZ. The NPCs can migrate to the lesioned area and may differentiate into neurons to support neurorepair. In our study, BrdU was chronically administered from days 8 to 20. Animals were euthanized on day 21. We found that delayed oral treatment with PF-00734,200 or sitagliptin increased BrdU labeling in lesioned striatum, but not in SVZ. It is hence likely that gliptins enhanced the migration of NPCs to the lesioned striatum. However, these cells did not express TH in striatum. The function of these BrdU (+) cells requires further investigation, particularly at times longer than 21 days post-lesioning, as in the present study, to allow these new cells more time to express their phenotypic markers. Of note, although quantifying the temporal and spatial expression of BrdU immunoreactivity is widely used in the evaluation of neurogenesis, several caveats exist with the use of this technique [86]. BrdU is marker of DNA synthesis, rather than cell proliferation, and hence it must be considered that BrdU immunohistochemistry not only detects newly dividing cells within the brain, but also cells potentially undergoing DNA repair or abortive cell cycle reentry.

Finally, in the light of the promising efficacy of clinically translatable oral doses of sitagliptin in this study, and the preservation of the GLP-1 and GIP receptors across age, and in rodent and human PD, evaluation of sitagliptin is warranted in female 6-OHDA challenged rats, as well as larger animal models. This is needed to determine whether the drug can achieve similar levels of DPP-4 inhibition and incretin elevations associated with efficacy in the present study. Prior human studies have demonstrated the efficacy and safety of sitagliptin across genders in T2DM [87] , and that there is no clinically meaningful effect of age or gender on its pharmacokinetics [88] .

**Conclusion**

Sitagliptin (Januvia) is one of 4 FDA approved, well-tolerated and widely used drugs in T2DM. In general, this class of drug is not associated with hypoglycemia, allowing use in normoglycemic non-diabetics. The gliptins’ proven pharmacological action is to inhibit the activity of DPP-4 and, thereby, to elevate the levels of the endogenous incretins GLP-1 and GIP [4, 5]. The beneficial neuroprotective and neuroregenerative actions at two clinically translational doses and route of administration in our 6-OHDA rat model of PD, strongly support the further evaluation of this class of drug as a new treatment strategy for PD. This view is substantiated by the known role of DPP-4 in inflammation [89-91], the involvement of inflammation in PD progression and aging [19, 92-96], the maintenance of the drug targets GLP-1R and GIPR across age and in PD brain, and the efficacy of the GLP-1R agonist exenatide in phase 2 clinical trials in moderate PD [28-30].

**Declarations**

**Ethical Approval**

**Institutional Review Board Statement:** The use of animals within this study was approved by the Animal Care and Use Committees of NIA and NIDA, National Institutes of Health, USA (Protocol #331-TGB-24 and #09-CNRB-9), and by the Animal Care and Use Committee, National Health Research Institutes, Taiwan (Protocol # 102068-A; 102102-A).

**Consent for publication**

**Not applicable (**the manuscript does not contain any individual person’s data in any form).

**Availability of Data and Material**

The datasets used and/or analyzed during the current study are available from the corresponding authors on reasonable request.

**Competing Interest**

The authors declare that they have no competing interests.

**Funding**

This research was supported in part by (i) the Michael J Fox Foundation (NYC, NY, USA), (ii) the Intramural Research Program of the National Institute on Aging, NIH (Baltimore, MD, USA): AG000333, (iii) the National Health Research Institutes, Taiwan and (iv) Ministry of Science and Technology, Taiwan (MOST-110-2320-B-400-007 and MOST-111-2320-B-400-010).

**Author Contributions**

Conception: MAT, MMZ, NHG, YW, BJH. Methodology: 6-OHDA lesions in rats S-JY, HS; Rat behavioral evaluations E-KB, S-JY; Preparation of tissue from rats S-JY, E-KB, HS, YL, NHG, YW; Rat immunohistochemistry/dopamine evaluation S-JY, E-KB, HS; Drug in food preparation YL, NHG, YW. Investigation: Mouse studies NHG, YL; Human PD tissue study KS, YL; Incretin/neuroinflammatory panel measures YL; Rat pharmacokinetic studies: MMZ, MAT, NHG, YL, HS. Resources: MMZ, MAT, KS; Data curation: S-JY, HS, E-KB, YL. Data analyses: YW, BJH, NHG, MMZ, MAT. Writing – original draft preparation: NHG, BJH, YW; Writing – review and editing: MMZ, MAT, KS, S-JY, E-KB, HS, YL. Visualization: S-JY, E-KB, YW, YL. Supervision: YW, NHG. Project Administration: YW, NHG, BJH, MAT, MMZ. Funding acquisition: NHG, YW, BJH, MAT, MMZ.

**Acknowledgments**

The authors thank the Michael J Fox Foundation for their support and scientific input, and Brian Goetz for advice on experimental design. MAT and MMZ formerly worked for Pfizer, and are grateful for the support of the company when in their prior positions. PF-00734200 was generously provided by Pfizer.

**References**

1. Organization. WH. Launch of WHO's Parkinson disease technical brief. . In; 2022.

2. Armstrong MJ, Okun MS. Diagnosis and Treatment of Parkinson Disease: A Review. JAMA. 2020;323(6):548-60.

3. Marino BLB, de Souza LR, Sousa KPA, Ferreira JV, Padilha EC, da Silva C, et al. Parkinson's Disease: A Review from Pathophysiology to Treatment. Mini Rev Med Chem. 2020;20(9):754-67.

4. Campos C, Unger J. Primary care management of type 2 diabetes: a comparison of the efficacy and safety of glucagon-like peptide-1 receptor agonists and dipeptidyl peptidase-4 inhibitors. Postgrad Med. 2021;133(8):843-53.

5. Gilbert MP, Pratley RE. GLP-1 Analogs and DPP-4 Inhibitors in Type 2 Diabetes Therapy: Review of Head-to-Head Clinical Trials. Front Endocrinol (Lausanne). 2020;11:178.

6. Baggio LL, Drucker DJ. Glucagon-like peptide-1 receptor co-agonists for treating metabolic disease. Mol Metab. 2021;46:101090.

7. Drucker DJ. Mechanisms of Action and Therapeutic Application of Glucagon-like Peptide-1. Cell Metab. 2018;27(4):740-56.

8. Nauck MA, Quast DR, Wefers J, Meier JJ. GLP-1 receptor agonists in the treatment of type 2 diabetes - state-of-the-art. Mol Metab. 2021;46:101102.

9. Holst JJ, Gasbjerg LS, Rosenkilde MM. The Role of Incretins on Insulin Function and Glucose Homeostasis. Endocrinology. 2021;162(7).

10. Tolhurst G, Reimann F, Gribble FM. Nutritional regulation of glucagon-like peptide-1 secretion. J Physiol. 2009;587(1):27-32.

11. Kaplan AM, Vigna SR. Gastric inhibitory polypeptide (GIP) binding sites in rat brain. Peptides. 1994;15(2):297-302.

12. Ludwig MQ, Todorov PV, Egerod KL, Olson DP, Pers TH. Single-Cell Mapping of GLP-1 and GIP Receptor Expression in the Dorsal Vagal Complex. Diabetes. 2021;70(9):1945-55.

13. Paratore S, Ciotti MT, Basille M, Vaudry D, Gentile A, Parenti R, et al. Gastric inhibitory polypeptide and its receptor are expressed in the central nervous system and support neuronal survival. Cent Nerv Syst Agents Med Chem. 2011;11(3):210-22.

14. Daniels D, Mietlicki-Baase EG. Glucagon-Like Peptide 1 in the Brain: Where Is It Coming From, Where Is It Going? Diabetes. 2019;68(1):15-7.

15. Kastin AJ, Akerstrom V, Pan W. Interactions of glucagon-like peptide-1 (GLP-1) with the blood-brain barrier. J Mol Neurosci. 2002;18(1-2):7-14.

16. Samms RJ, Coghlan MP, Sloop KW. How May GIP Enhance the Therapeutic Efficacy of GLP-1? Trends Endocrinol Metab. 2020;31(6):410-21.

17. Diz-Chaves Y, Mastoor Z, Spuch C, Gonzalez-Matias LC, Mallo F. Anti-Inflammatory Effects of GLP-1 Receptor Activation in the Brain in Neurodegenerative Diseases. Int J Mol Sci. 2022;23(17).

18. Holscher C. Protective properties of GLP-1 and associated peptide hormones in neurodegenerative disorders. Br J Pharmacol. 2022;179(4):695-714.

19. Kopp KO, Glotfelty EJ, Li Y, Greig NH. Glucagon-like peptide-1 (GLP-1) receptor agonists and neuroinflammation: Implications for neurodegenerative disease treatment. Pharmacol Res. 2022;186:106550.

20. Foltynie T, Athauda D. Repurposing anti-diabetic drugs for the treatment of Parkinson's disease: Rationale and clinical experience. Prog Brain Res. 2020;252:493-523.

21. Girges C, Vijiaratnam N, Athauda D, Auld G, Gandhi S, Foltynie T. The Future of Incretin-Based Approaches for Neurodegenerative Diseases in Older Adults: Which to Choose? A Review of their Potential Efficacy and Suitability. Drugs Aging. 2021;38(5):355-73.

22. Glotfelty EJ, Olson L, Karlsson TE, Li Y, Greig NH. Glucagon-like peptide-1 (GLP-1)-based receptor agonists as a treatment for Parkinson's disease. Expert Opin Investig Drugs. 2020;29(6):595-602.

23. Holscher C. Glucagon-like peptide 1 and glucose-dependent insulinotropic peptide hormones and novel receptor agonists protect synapses in Alzheimer's and Parkinson's diseases. Front Synaptic Neurosci. 2022;14:955258.

24. Nowell J, Blunt E, Edison P. Incretin and insulin signaling as novel therapeutic targets for Alzheimer's and Parkinson's disease. Mol Psychiatry. 2023;28(1):217-29.

25. Reich N, Holscher C. The neuroprotective effects of glucagon-like peptide 1 in Alzheimer's and Parkinson's disease: An in-depth review. Front Neurosci. 2022;16:970925.

26. Aviles-Olmos I, Dickson J, Kefalopoulou Z, Djamshidian A, Ell P, Soderlund T, et al. Exenatide and the treatment of patients with Parkinson's disease. J Clin Invest. 2013;123(6):2730-6.

27. Aviles-Olmos I, Dickson J, Kefalopoulou Z, Djamshidian A, Kahan J, Ell P, et al. Motor and cognitive advantages persist 12 months after exenatide exposure in Parkinson's disease. J Parkinsons Dis. 2014;4(3):337-44.

28. Athauda D, Gulyani S, Karnati HK, Li Y, Tweedie D, Mustapic M, et al. Utility of Neuronal-Derived Exosomes to Examine Molecular Mechanisms That Affect Motor Function in Patients With Parkinson Disease: A Secondary Analysis of the Exenatide-PD Trial. JAMA Neurol. 2019;76(4):420-9.

29. Athauda D, Maclagan K, Budnik N, Zampedri L, Hibbert S, Aviles-Olmos I, et al. Post hoc analysis of the Exenatide-PD trial-Factors that predict response. Eur J Neurosci. 2019;49(3):410-21.

30. Athauda D, Maclagan K, Skene SS, Bajwa-Joseph M, Letchford D, Chowdhury K, et al. Exenatide once weekly versus placebo in Parkinson's disease: a randomised, double-blind, placebo-controlled trial. Lancet. 2017;390(10103):1664-75.

31. Deacon CF. Metabolism of GIP and the contribution of GIP to the glucose-lowering properties of DPP-4 inhibitors. Peptides. 2020;125:170196.

32. Holst JJ. From the Incretin Concept and the Discovery of GLP-1 to Today's Diabetes Therapy. Front Endocrinol (Lausanne). 2019;10:260.

33. Reagan-Shaw S, Nihal M, Ahmad N. Dose translation from animal to human studies revisited. FASEB J. 2008;22(3):659-61.

34. Yin LH, Shen H, Diaz-Ruiz O, Backman CM, Bae E, Yu SJ, et al. Early post-treatment with 9-cis retinoic acid reduces neurodegeneration of dopaminergic neurons in a rat model of Parkinson's disease. BMC Neurosci. 2012;13:120.

35. Li Y, Perry T, Kindy MS, Harvey BK, Tweedie D, Holloway HW, et al. GLP-1 receptor stimulation preserves primary cortical and dopaminergic neurons in cellular and rodent models of stroke and Parkinsonism. Proc Natl Acad Sci U S A. 2009;106(4):1285-90.

36. Harvey BK, Mark A, Chou J, Chen GJ, Hoffer BJ, Wang Y. Neurotrophic effects of bone morphogenetic protein-7 in a rat model of Parkinson's disease. Brain Res. 2004;1022(1-2):88-95.

37. Hudson JL, van Horne CG, Stromberg I, Brock S, Clayton J, Masserano J, et al. Correlation of apomorphine- and amphetamine-induced turning with nigrostriatal dopamine content in unilateral 6-hydroxydopamine lesioned rats. Brain Res. 1993;626(1-2):167-74.

38. Ungerstedt U. 6-hydroxydopamine-induced degeneration of the nigrostriatal dopamine pathway: the turning syndrome. Pharmacol Ther B. 1976;2(1):37-40.

39. Ungerstedt U, Ljungberg T, Hoffer B, Siggins G. Dopaminergic supersensitivity in the striatum. Adv Neurol. 1975;9:57-65.

40. Ungerstedt U, Ljungberg T, Steg G. Behavioral, physiological, and neurochemical changes after 6-hydroxydopamine-induced degeneration of the nigro-striatal dopamine neurons. Adv Neurol. 1974;5:421-6.

41. Lundblad MP, D.A.; Burmeister, J.J.; Quintero, J.E.; Huettl, P.; Pomerleau, F.; Zahniser, N.R.; Gerhardt, G.A. Tonic and phasic amperometric monitoring of dopamine using microelectrode arrays in rat striatum. Appl Sci 2020;10.

42. Chen S, Yu SJ, Li Y, Lecca D, Glotfelty E, Kim HK, et al. Post-treatment with PT302, a long-acting Exendin-4 sustained release formulation, reduces dopaminergic neurodegeneration in a 6-Hydroxydopamine rat model of Parkinson's disease. Sci Rep. 2018;8(1):10722.

43. Kaminska K, Lenda T, Konieczny J, Lorenc-Koci E. Behavioral and neurochemical interactions of the tricyclic antidepressant drug desipramine with L-DOPA in 6-OHDA-lesioned rats. Implications for motor and psychiatric functions in Parkinson's disease. Psychopharmacology (Berl). 2022;239(11):3633-56.

44. Lenda T, Ossowska K, Berghauzen-Maciejewska K, Matloka M, Pieczykolan J, Wieczorek M, et al. Antiparkinsonian-like effects of CPL500036, a novel selective inhibitor of phosphodiesterase 10A, in the unilateral rat model of Parkinson's disease. Eur J Pharmacol. 2021;910:174460.

45. Liu DM, Lin SZ, Wang SD, Wu MY, Wang Y. Xenografting human T2 sympathetic ganglion from hyperhidrotic patients provides short-term restoration of catecholaminergic functions in hemiparkinsonian athymic rats. Cell Transplant. 1999;8(6):583-91.

46. Luo Y HB, Wang Y Rotation, Drug-induced. . Encyclopedia of Movement Disorders (MOVE). 2010:49-51.

47. Krasnova IN, Bychkov ER, Lioudyno VI, Zubareva OE, Dambinova SA. Intracerebroventricular administration of substance P increases dopamine content in the brain of 6-hydroxydopamine-lesioned rats. Neuroscience. 2000;95(1):113-7.

48. Scheen AJ. Pharmacokinetics of dipeptidylpeptidase-4 inhibitors. Diabetes Obes Metab. 2010;12(8):648-58.

49. Zeng W, Xu Y, Constanzer M, Woolf EJ. Determination of sitagliptinin human plasma using protein precipitation and tandem mass spectrometry. J Chromatogr B Analyt Technol Biomed Life Sci. 2010;878(21):1817-23.

50. Sharma R, Sun H, Piotrowski DW, Ryder TF, Doran SD, Dai H, et al. Metabolism, excretion, and pharmacokinetics of ((3,3-difluoropyrrolidin-1-yl)((2S,4S)-4-(4-(pyrimidin-2-yl)piperazin-1-yl)pyrrolidin-2-yl)methanone, a dipeptidyl peptidase inhibitor, in rat, dog and human. Drug Metab Dispos. 2012;40(11):2143-61.

51. Tamargo IA, Bader M, Li Y, Yu SJ, Wang Y, Talbot K, et al. Novel GLP-1R/GIPR co-agonist "twincretin" is neuroprotective in cell and rodent models of mild traumatic brain injury. Exp Neurol. 2017;288:176-86.

52. Yang X, Feng P, Ji R, Ren Y, Wei W, Holscher C. Therapeutic application of GLP-1 and GIP receptor agonists in Parkinson's disease. Expert Opin Ther Targets. 2022;26(5):445-60.

53. Kovalevich J, Santerre M, Langford D. Considerations for the Use of SH-SY5Y Neuroblastoma Cells in Neurobiology. Methods Mol Biol. 2021;2311:9-23.

54. Mulvihill EE, Drucker DJ. Pharmacology, physiology, and mechanisms of action of dipeptidyl peptidase-4 inhibitors. Endocr Rev. 2014;35(6):992-1019.

55. Hong WJ, Petell JK, Swank D, Sanford J, Hixson DC, Doyle D. Expression of dipeptidyl peptidase IV in rat tissues is mainly regulated at the mRNA levels. Exp Cell Res. 1989;182(1):256-66.

56. Fuchs H, Binder R, Greischel A. Tissue distribution of the novel DPP-4 inhibitor BI 1356 is dominated by saturable binding to its target in rats. Biopharm Drug Dispos. 2009;30(5):229-40.

57. Barnes K, Kenny AJ, Turner AJ. Localization of aminopeptidase N and dipeptidyl peptidase IV in pig striatum and in neuronal and glial cell cultures. Eur J Neurosci. 1994;6(4):531-7.

58. Bourne A, Barnes K, Taylor BA, Turner AJ, Kenny AJ. Membrane peptidases in the pig choroid plexus and on other cell surfaces in contact with the cerebrospinal fluid. Biochem J. 1989;259(1):69-80.

59. Rohnert P, Schmidt W, Emmerlich P, Goihl A, Wrenger S, Bank U, et al. Dipeptidyl peptidase IV, aminopeptidase N and DPIV/APN-like proteases in cerebral ischemia. J Neuroinflammation. 2012;9:44.

60. Kiraly K, Kozsurek M, Lukacsi E, Barta B, Alpar A, Balazsa T, et al. Glial cell type-specific changes in spinal dipeptidyl peptidase 4 expression and effects of its inhibitors in inflammatory and neuropatic pain. Sci Rep. 2018;8(1):3490.

61. Bertilsson G, Patrone C, Zachrisson O, Andersson A, Dannaeus K, Heidrich J, et al. Peptide hormone exendin-4 stimulates subventricular zone neurogenesis in the adult rodent brain and induces recovery in an animal model of Parkinson's disease. J Neurosci Res. 2008;86(2):326-38.

62. Harkavyi A, Abuirmeileh A, Lever R, Kingsbury AE, Biggs CS, Whitton PS. Glucagon-like peptide 1 receptor stimulation reverses key deficits in distinct rodent models of Parkinson's disease. J Neuroinflammation. 2008;5:19.

63. Jeong SH, Chung SJ, Yoo HS, Hong N, Jung JH, Baik K, et al. Beneficial effects of dipeptidyl peptidase-4 inhibitors in diabetic Parkinson's disease. Brain. 2021;144(4):1127-37.

64. Brauer R, Wei L, Ma T, Athauda D, Girges C, Vijiaratnam N, et al. Diabetes medications and risk of Parkinson's disease: a cohort study of patients with diabetes. Brain. 2020;143(10):3067-76.

65. Svenningsson P, Wirdefeldt K, Yin L, Fang F, Markaki I, Efendic S, et al. Reduced incidence of Parkinson's disease after dipeptidyl peptidase-4 inhibitors-A nationwide case-control study. Mov Disord. 2016;31(9):1422-3.

66. Bader M, Li Y, Tweedie D, Shlobin NA, Bernstein A, Rubovitch V, et al. Neuroprotective Effects and Treatment Potential of Incretin Mimetics in a Murine Model of Mild Traumatic Brain Injury. Front Cell Dev Biol. 2019;7:356.

67. Li Y, Glotfelty EJ, Namdar I, Tweedie D, Olson L, Hoffer BJ, et al. Neurotrophic and neuroprotective effects of a monomeric GLP-1/GIP/Gcg receptor triagonist in cellular and rodent models of mild traumatic brain injury. Exp Neurol. 2020;324:113113.

68. Tai J, Liu W, Li Y, Li L, Holscher C. Neuroprotective effects of a triple GLP-1/GIP/glucagon receptor agonist in the APP/PS1 transgenic mouse model of Alzheimer's disease. Brain Res. 2018;1678:64-74.

69. Bergman A, Ebel D, Liu F, Stone J, Wang A, Zeng W, et al. Absolute bioavailability of sitagliptin, an oral dipeptidyl peptidase-4 inhibitor, in healthy volunteers. Biopharm Drug Dispos. 2007;28(6):315-22.

70. Herman GA, Mistry GC, Yi B, Bergman AJ, Wang AQ, Zeng W, et al. Evaluation of pharmacokinetic parameters and dipeptidyl peptidase-4 inhibition following single doses of sitagliptin in healthy, young Japanese males. Br J Clin Pharmacol. 2011;71(3):429-36.

71. Herman GA, Stevens C, Van Dyck K, Bergman A, Yi B, De Smet M, et al. Pharmacokinetics and pharmacodynamics of sitagliptin, an inhibitor of dipeptidyl peptidase IV, in healthy subjects: results from two randomized, double-blind, placebo-controlled studies with single oral doses. Clin Pharmacol Ther. 2005;78(6):675-88.

72. Chu XY, Bleasby K, Yabut J, Cai X, Chan GH, Hafey MJ, Xu S, Bergman AJ, Braun MP, Dean DC, Evers R. Transport of the dipeptidyl peptidase-4 inhibitor sitagliptin by human organic anion transporter 3, organic anion transporting polypeptide 4C1, and multidrug resistance P-glycoprotein. J Pharmacol Exp Ther 2007; 321:673-683. <https://doi.org/10.1124/jpet.106.116517>.

73. Avelar AJ, Juliano SA, Garris PA. Amphetamine augments vesicular dopamine release in the dorsal and ventral striatum through different mechanisms. J Neurochem. 2013;125(3):373-85.

74. Cadet JL, Brannock C, Krasnova IN, Ladenheim B, McCoy MT, Chou J, et al. Methamphetamine-induced dopamine-independent alterations in striatal gene expression in the 6-hydroxydopamine hemiparkinsonian rats. PLoS One. 2010;5(12):e15643.

75. Badawi GA, Abd El Fattah MA, Zaki HF, El Sayed MI. Sitagliptin and liraglutide reversed nigrostriatal degeneration of rodent brain in rotenone-induced Parkinson's disease. Inflammopharmacology. 2017;25(3):369-82.

76. Badawi GA, Abd El Fattah MA, Zaki HF, El Sayed MI. Sitagliptin and Liraglutide Modulate L-dopa Effect and Attenuate Dyskinetic Movements in Rotenone-Lesioned Rats. Neurotox Res. 2019;35(3):635-53.

77. Abdelsalam RM, Safar MM. Neuroprotective effects of vildagliptin in rat rotenone Parkinson's disease model: role of RAGE-NFkappaB and Nrf2-antioxidant signaling pathways. J Neurochem. 2015;133(5):700-7.

78. Sayed NH, Fathy N, Kortam MA, Rabie MA, Mohamed AF, Kamel AS. Vildagliptin Attenuates Huntington's Disease through Activation of GLP-1 Receptor/PI3K/Akt/BDNF Pathway in 3-Nitropropionic Acid Rat Model. Neurotherapeutics. 2020;17(1):252-68.

79. Nassar NN, Al-Shorbagy MY, Arab HH, Abdallah DM. Saxagliptin: a novel antiparkinsonian approach. Neuropharmacology. 2015;89:308-17.

80. Safar MM, Abdelkader NF, Ramadan E, Kortam MA, Mohamed AF. Novel mechanistic insights towards the repositioning of alogliptin in Parkinson's disease. Life Sci. 2021;287:120132.

81. Maanvi, Kumari S, Deshmukh R. Dipeptidyl peptidase 4(DPP4) inhibitors stride up the management of Parkinson's disease. Eur J Pharmacol. 2023;939:175426.

82. Darsalia V, Olverling A, Larsson M, Mansouri S, Nathanson D, Nystrom T, et al. Linagliptin enhances neural stem cell proliferation after stroke in type 2 diabetic mice. Regul Pept. 2014;190-191:25-31.

83. Gault VA, Lennox R, Flatt PR. Sitagliptin, a dipeptidyl peptidase-4 inhibitor, improves recognition memory, oxidative stress and hippocampal neurogenesis and upregulates key genes involved in cognitive decline. Diabetes Obes Metab. 2015;17(4):403-13.

84. Abe T, Aburakawa D, Niizuma K, Iwabuchi N, Kajitani T, Wakao S, et al. Intravenously Transplanted Human Multilineage-Differentiating Stress-Enduring Cells Afford Brain Repair in a Mouse Lacunar Stroke Model. Stroke. 2020;51(2):601-11.

85. Kaneko Y, Lee JY, Tajiri N, Tuazon JP, Lippert T, Russo E, et al. Translating intracarotid artery transplantation of bone marrow-derived NCS-01 cells for ischemic stroke: Behavioral and histological readouts and mechanistic insights into stem cell therapy. Stem Cells Transl Med. 2020;9(2):203-20.

86. Taupin P. BrdU immunohistochemistry for studying adult neurogenesis: paradigms, pitfalls, limitations, and validation. Brain Res Rev. 2007; 53(1):198-214.

87. Zhan M, Xu T, Wu F, Tang Y. Sitagliptin in the treatment of type 2 diabetes: a meta-analysis. J Evid Based Med. 2012;5(3):154-65.

88. Bergman A SC, Zhou YY, Laethem M, Lachaert R, Snyder K, Hilliard D, Tanaka W, Zeng W, Wang A, Chen L, Winchell G, Ramael S, Davies M, Wagner J, Herman G. . Lack of a clinically meaningful effect on age, gender or obesity on the pharmacokinetics of the DPP4 inhibitor MK-0431. . Diabetes. 2005;54:A506.

89. Atkin SL, Katsiki N, Banach M, Mikhailidis DP, Pirro M, Sahebkar A. Effect of dipeptidyl peptidase-4 inhibitors on circulating tumor necrosis factor-alpha concentrations: A systematic review and meta-analysis of controlled trials. J Diabetes Complications. 2017;31(9):1458-64.

90. Klemann C, Wagner L, Stephan M, von Horsten S. Cut to the chase: a review of CD26/dipeptidyl peptidase-4's (DPP4) entanglement in the immune system. Clin Exp Immunol. 2016;185(1):1-21.

91. Trzaskalski NA, Fadzeyeva E, Mulvihill EE. Dipeptidyl Peptidase-4 at the Interface Between Inflammation and Metabolism. Clin Med Insights Endocrinol Diabetes. 2020;13:1179551420912972.

92. Joers V, Tansey MG, Mulas G, Carta AR. Microglial phenotypes in Parkinson's disease and animal models of the disease. Prog Neurobiol. 2017;155:57-75.

93. Kuter KZ, Cenci MA, Carta AR. The role of glia in Parkinson's disease: Emerging concepts and therapeutic applications. Prog Brain Res. 2020;252:131-68.

94. Marogianni C, Sokratous M, Dardiotis E, Hadjigeorgiou GM, Bogdanos D, Xiromerisiou G. Neurodegeneration and Inflammation-An Interesting Interplay in Parkinson's Disease. Int J Mol Sci. 2020;21(22).

95. Mulas G, Espa E, Fenu S, Spiga S, Cossu G, Pillai E, et al. Differential induction of dyskinesia and neuroinflammation by pulsatile versus continuous l-DOPA delivery in the 6-OHDA model of Parkinson's disease. Exp Neurol. 2016;286:83-92.

96. Pajares M, A IR, Manda G, Bosca L, Cuadrado A. Inflammation in Parkinson's Disease: Mechanisms and Therapeutic Implications. Cells. 2020;9(7).

**Supplemental Figures**

**S Fig 1.**

**Supplemental Fig 1: Chronic treatment with PF-00734,200 (PF) or sitagliptin (Sita) did not alter body weight in 6‐OHDA lesioned animals**. Animals received vehicle (Veh), PF or sita (12 mg/kg daily) from days 7 after 6‐OHDA lesioning. Body weight was examined one day before and 28 days after 6‐OHDA lesioning (all values are means ± SEM). No difference was found among these drug treatments (One way ANOVA) (n≥8 across groups).

**S Fig 2.**

**Supplemental Fig 2: Direct brain (i.c.v.) administration of PF-00734,200 failed to reduce methamphetamine (meth)-induced rotation or provide neuroprotection in 6-OHDA-lesioned rats, in contrast to oral PF-00734,200.** (A) PF-00734,200 was administered directly into the brain (i.c.v.) immediately prior to a 6-OHDA unilateral lesion to define whether the neuroprotective action of PF-00734,200 (evident in Fig. 2 main manuscript text) was mediated via direct drug action, rather than a drug-induced elevation of systemic and central incretins. (B) Meth-induced rotation (evaluated on day 30) was not different between PF-00734,200 i.c.v. and vehicle i.c.v. treated animal groups. (C) Evaluation of TH staining in striatum and substantia nigra pars compacta (SNc) demonstrated a lack of neuroprotection in PF-00734,200 i.c.v. administered rats, in contrast to those administered the drug orally. All values are means ± SEM (n=8 vehicle (sterile physiological saline, 20 μL); n=6 PF-00735,200).

**S Fig 3.**

**
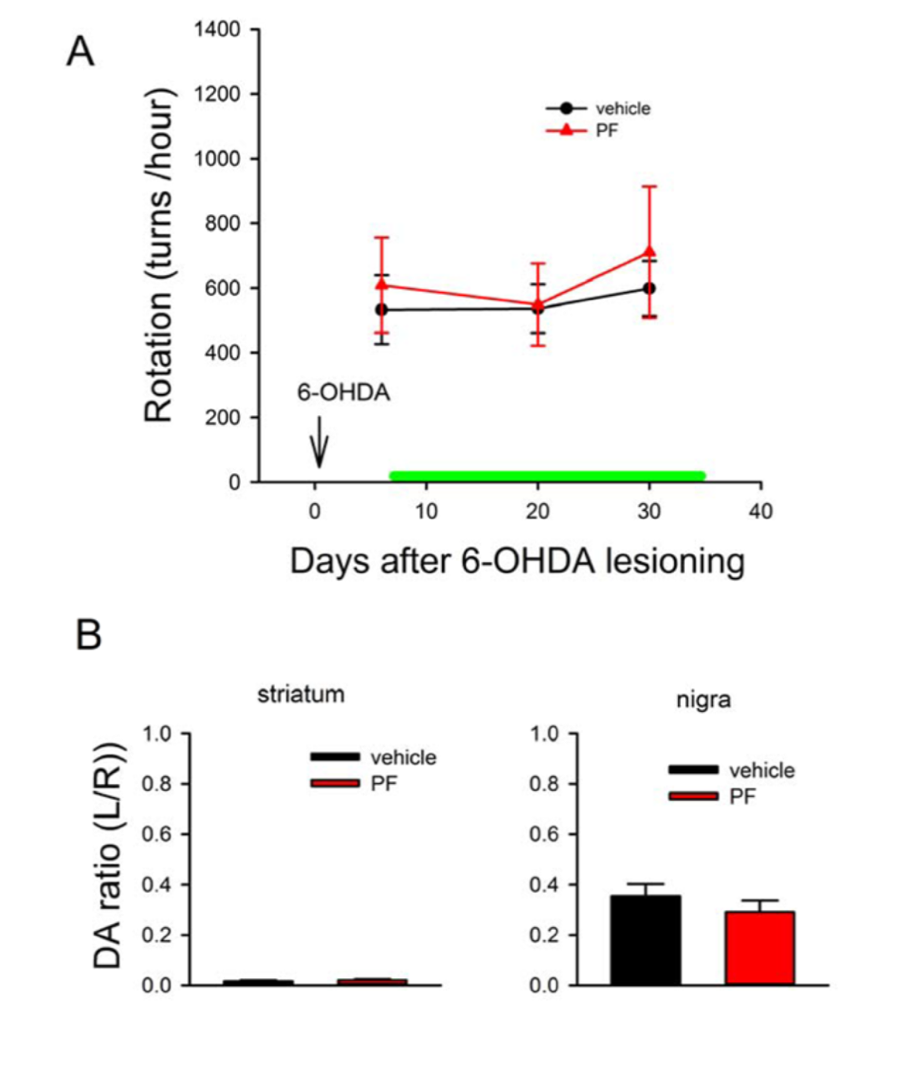
**

**Supplemental Fig 3: Delayed oral treatment with low dose PF‐00734,200 (PF) did not alter rotational behavior and DA levels in 6‐OHDA lesioned rats**. A 6-OHDA unilateral medial forebrain bundle lesion was performed on day 0. Thereafter, low dose of PF‐00734,200 (10 mg/kg daily) or vehicle was given daily from days 7 to 35(green line in (A)) after 6‐OHDA lesioning in 14 rats (n=7/group). (A) PF‐00734,200 did not alter rotational behavior (p=0.511, two‐way ANOVA). (B) Striatal and substantia nigra tissues were collected on day 35 post lesion. Delayed treatment with PF did not alter DA level in both areas (all values are means ± SEM; striatum, p=0.18; substantia nigra, p=0.17, t‐test).
